# The Effectiveness of Simulation‐Based Versus Patient‐Based Clinical Education in Nursing: A Systematic Review and Meta‐Analysis

**DOI:** 10.1002/nop2.70689

**Published:** 2026-09-04

**Authors:** Louise Prothero, Naim Abdulmohdi, Siân Shaw, Mary Edmonds, Catherine Meads

**Affiliations:** ^1^ School of Midwifery & Community Health, Anglia Ruskin University Chelmsford UK; ^2^ School of Nursing, Anglia Ruskin University Chelmsford UK; ^3^ Faculty of Health, Medicine and Social Care, Anglia Ruskin University Chelmsford UK

**Keywords:** clinical education, clinical training, meta‐analysis, nurse education, simulation education, systematic review

## Abstract

**Aim:**

To compare the effectiveness of simulation‐based education (alone or in combination with patient‐based clinical teaching) in undergraduate nursing students, when measured using objectively assessed outcomes.

**Design:**

Systematic review with meta‐analyses.

**Review Methods:**

Comparative studies in undergraduate nurses where outcomes of externally verified exams, proficiencies or competencies for both groups were included. Citation‐checking, data‐extraction and quality‐assessment (using CASP checklists) were by two reviewers independently, and Revman (5.4) was used for meta‐analysis, using standardised mean differences (SMD).

**Data Sources:**

Ten databases (platforms) were searched from January 2000 to January 2024: MEDLINE (OVID), Embase (OVID), Maternity and Childcare (OVID), CINAHL (EBSCO), PsycINFO (EBSCO), Central (Cochrane Library), Scopus, Science Citation Index (Web of Science), ERIC (EBSCO) and Assia (ProQuest).

**Results:**

Forty‐two studies (5336 participants) were included: 19 randomised controlled trials, two randomised crossover trials, three cohort and 18 case–control studies. Twenty‐eight studies compared simulation‐based education to clinical teaching, 11 compared simulation‐based education plus clinical teaching to clinical teaching only, and three compared more versus less simulation‐based education in courses. Comparison 1: 21 studies (*n* = 2329 participants) could be meta‐analysed, giving SMD = 0.96 (95% confidence intervals = 0.63–1.30). Comparison 2: 11 studies (*n* = 918 participants) gave SMD = 0.82 (95% confidence intervals = 0.49–1.15). All included studies reported as good or better results for simulation education.

**Conclusions:**

Simulation‐based education, alone or in combination, may be as good as patient‐based clinical teaching only to teach care skills to nursing students. Future simulation‐based education versus patient‐based clinical teaching evaluations should also measure clinical and patient‐based outcomes.

**Implications for the Profession and/or Patient Care:**

More simulation‐based education of nursing students may result in fewer patients used for teaching.

**Impact:**

Increased simulation‐based education may enhance undergraduate teaching, as more can be taught patient‐based clinical skills in an educational setting without apparently reducing educational performance.

**Patient or Public Contribution:**

No patient or public contribution as this is a systematic review.

**Trial Registration:**

PROSPERO (https://www.crd.york.ac.uk/prospero): CRD42022363205

## 1 Background

Simulation can be defined as the imitation of the operation of a real‐world process or system over time (Mourtzis et al. 2014). Several authors describe simulation as a technique rather than a technology, employed as an active learning strategy to replace or amplify real‐world experiences through guided, often immersive and fully interactive activities that evoke or replicate substantial aspects, or the entirety, of clinical situations. (Gaba 2004; Dieckmann et al. 2007; Abdulmohdi 2019; INACSL 2021).

Simulation in healthcare aims to mimic patient‐based clinical environments and aspects of face‐to‐face clinical care to improve healthcare provider performance, healthcare processes, and patient outcomes (Abdulmohdi 2019). The use of simulation‐based education aids the development of clinical knowledge, skills, and practice in students and increases the possibility of applying the acquired knowledge and skills into patient‐based clinical practice (Harrison et al. 2024). Unlike clinical practice with patients, where the needs of the patient are the main priorities, simulation exercises are centred upon the needs of the student and can be tailored to meet required learning outcomes. Many simulation‐based education teaching encounters use debriefing afterwards, which is a guided discussion with students to enable a clearer understanding of their thoughts, actions and consequences (Abdulmohdi 2019). Debriefing can be thought of as didactic transposition of experiential knowledge—the transformation of knowledge acquired through simulated experience into applied clinical practice and has been found to promote deeper learning and improve learning outcomes (Duff 2024; Fegran et al. 2023; INACSL Standards Committee et al. 2025).

Patient‐based teaching is often considered the gold standard for clinical skills training. In response to the COVID‐19 pandemic and the constrained clinical capacity for patient‐based placements, the UK Nursing and Midwifery Council (Nursing and Midwifery Council [NMC] 2022) increased the allowances for simulation practice learning hours in university settings from 300 to 600 clinical hours. Although simulation‐based education is commonly employed in nursing curricula, there exist notable disparities in the practice hours mandated by nursing courses globally (Garrow et al. 2022) and in how simulation‐based education is incorporated into these curricula (Poston et al. 2025). UK Nursing programmes are currently under heightened scrutiny to produce graduates capable of delivering safe and effective care, underscoring the need for further research to ensure that simulation‐based education can effectively replace clinical practice to teach nurses so that they are fit to practice at the point of registration (NMC 2023). High‐fidelity simulations can enhance learning outcomes, but they must be carefully designed to align with the specific competencies being taught (Kim et al. 2016; Ng et al. 2021).

There are large numbers of published studies evaluating simulation‐based education in nurses (Rutherford‐Hemming and Alfes 2017), many of which provide simulation learning activities to a single group of students and then assess them afterwards for educational success. Evaluations are sometimes enhanced by using a pretest‐post‐test design. However, this approach to evaluating an educational intervention cannot directly compare the effectiveness of simulation‐based education against other instructional methods. In order to compare the effectiveness of two modes of education, a more robust comparative design is needed that involves two or more groups of students, where one group receives simulation‐based education while others are taught using traditional methods in various settings, such as in classrooms or patient‐based clinical environments (Thomas and Barker 2022). This comparative approach allows for a more comprehensive evaluation of teaching modes using self‐assessed and objectively assessed outcomes, such as competency, anxiety levels, Objective Structured Clinical Examination (OSCE) results, and overall acceptability of the teaching methods employed (Cook et al. 2012; Eppich and Reedy 2022; McGaghie et al. 2010).

If simulation is to be used to teach clinical skills, there is a need for rigorous comparative studies of simulation education and patient‐based education on the same set of clinical skills, to ensure that student nurses attain equivalent competencies with the two modes of teaching. Until now, the evidence has not been conclusive regarding whether nurses taught through simulation‐based education will gain similar or better competency in learning outcomes as those taught the same topics through clinical patient‐based teaching.

Kirkpatrick (1967) developed a 4‐level model on learning from training programmes. The levels are (1) Reactions, (2) Learning, (3) Behaviour and (4) Results—effects or ultimate outcomes, such as patient outcomes, safety, organisational impact or other measurable benefits resulting from changed behaviours. Studies consistently report that while lower Kirkpatrick levels (1–3) show positive outcomes, the assessment of Level 4 benefits is much less documented, highlighting a significant gap in evaluation practices in healthcare education (Delisle et al. 2019).

A brief search of published systematic reviews and meta‐analyses included in one of the academic journals that specialises in simulation education, called Clinical Simulation in Nursing, by one of the authors (CM), (by putting the search term systematic review and then meta‐analysis in the journal search bar) revealed 147 systematic review articles and then 66 meta‐analysis articles since inception of the journal (2008). The abstracts of each were read and none fully answered our research question.

Systematic reviews closest to answering this question were an early systematic review of simulation in preparation or substitution for clinical placement (Larue et al. 2015), a realist meta‐narrative review of simulation to replace clinical hours in nursing (Roberts et al. 2019), and a review of hospital‐based simulation in nursing education (Rutherford‐Hemming and Alfes 2017). Larue et al. (2015) included 33 clinical studies, systematic reviews, and guidelines published between 2008 and 2014, but there is no list of included studies, and it is difficult to know how some studies contributed to different parts of the review. The section on substitution for clinical placement principally discussed the issue of throughput of nurses in clinical education and the possibility of using simulation to replace some of the patient‐based clinical hours. It also touched on the clinical effectiveness of teaching, but the only evidence presented was the randomised controlled trial by Hayden et al. (2014). Rutherford‐Hemming and Alfes (2017) included studies in English and published between 2012 and 2015 that evaluated any form of hospital‐based simulation‐based education. The review included 65 studies of various designs but did not evaluate the effectiveness of simulation‐based education in hospital settings compared to patient‐based teaching. Roberts et al. (2019) conducted a meta‐narrative review to evaluate whether simulation‐based education can replace clinical hours when teaching undergraduate nurses. It included 12 studies published in English between 2007 and 2018 and conducted quality assessment, but no meta‐analysis due to the heterogeneity of approaches to simulation‐based education and outcome measures. The meta‐synthesis found that:

there was no significant difference in learning or student outcomes between simulation and clinical practice, when a percentage of clinical placement hours was replaced with simulation.

It is unclear why Roberts et al. (2019) did not include a number of studies published during the eligible timeframe (e.g., Centrella‐Nigro et al. 2016; Hall 2015; Hansen and Bratt 2017; Harris 2011; Luctkar‐Flude et al. 2012; Sears et al. 2010; Witt et al. 2018; Yu and Kang 2017), as these seem to match the stated inclusion criteria. It is unclear whether or not the addition of these studies would have changed their conclusions. Since 2018, a number of additional studies have been published on our research question (see this systematic review lists of included studies).

This current systematic review determines the effectiveness of simulated learning (alone or in combination with patient‐based clinical education) to teach clinical practice‐based patient care skills to nursing students, compared to clinical practice‐based and/or patient‐based teaching only on the same topic, using objectively measured assessment outcomes for both groups.

## 2 Methods

A protocol for this systematic review was registered with the PROSPERO database (https://www.crd.york.ac.uk/prospero). Results are reported in accordance with the Preferred Reporting Items for Systematic Reviews and Meta‐Analyses (PRISMA) guideline (Moher et al. 2009).

### 2.1 Inclusion and Exclusion Criteria

Participants were undergraduate students studying for any nursing qualification at any educational or health‐related establishment, including hospitals, primary care providers, universities or training colleges anywhere in the world. Interventions were clinically based topics taught through any form of simulation‐based education. Simulation was defined as ‘an educational method which uses a variety of modalities to support students in developing their knowledge, behaviours and skills, with the opportunity for repetition, feedback, evaluation and reflection to achieve their programme outcomes and be confirmed as capable of safe and effective practice’ (NMC 2023). Comparators were the same set of skills and abilities being taught using face‐to‐face clinical practice‐based and/or patient‐based teaching. Studies were only included if they reported one or more outcomes: (1) any relevant clinical outcomes (e.g., mortality, wound infection, length of hospital stay), (2) patient‐based care being given appropriately, (3) exam success, OSCE success, assignment success, externally verified proficiency or competency, teacher verified proficiency or competency, (4) patient acceptability of nursing care given, and (5) staff resource time for teaching of the students. Effect measures could be given in a variety of ways, such as exam pass‐rate percentages, mean scores on competency assessment or patient ratings of nursing care. Included were any empirical studies with a concurrent comparator, including cluster, cross‐over or standard randomised controlled trials (RCT), cohort, or case–control studies. Publications could be in any language and must have been fully published in a peer‐reviewed journal between January 2000 and January 2024. The year 2000 was chosen so that more current, up‐to‐date studies were used and because simulation in health‐related education was starting to be recommended at the start of the new millennium (Department of Health 2001). Excluded were conference abstracts, single group studies with a historical control (i.e., students assessed before and after an episode of simulation teaching) and studies with mixed participant groups (e.g., nursing students and medical students). Self‐assessed outcomes were excluded because they may not be accurate (Garner et al. 2020; Alastalo et al. 2022).

### 2.2 Search Strategy

Searches were performed in 10 databases (with the platforms they were searched on): MEDLINE (OVID), Embase (OVID), Maternity and Childcare (OVID), CINAHL (EBSCO), PsycINFO (EBSCO), Central (Cochrane Library), Scopus, Science Citation Index (Web of Science), ERIC (EBSCO) and Assia (ProQuest), from 2000 to 2024. The search strategy was based on synonyms of ‘simulation’, ‘assessment’, ‘teaching’, ‘nurse’, ‘undergraduate’, ‘trial’ and ‘patient’. Searches combined free text words and Medical Subject Headings (MeSH) terms which were exploded to gain maximum capture. The search strategy, originally for MEDLINE (OVID) (see Data S1), was adapted for use with the other databases. Some nursing journals are not indexed in the 10 databases searched, so studies included in 17 systematic reviews found through a Google search were checked for eligibility. The journal Clinical Simulation in Nursing was searched for systematic reviews and meta‐analyses published from January 2000 onwards, which were then sifted for eligible studies. Reference lists of included studies were searched for additional includable studies.

### 2.3 Citation Checking, Data Extraction and Quality Assessment

Two researchers (LP, CM) evaluated whether each study met the inclusion criteria, and disagreements were resolved through discussion. Data extraction was onto tables in the Microsoft Word package. Quality assessment was assessed using Critical Appraisal Skills Programme (CASP) checklists (Critical Appraisal Skills Programme [CASP] 2023) appropriate to the study design of each study. Data extraction and quality assessment were by one reviewer (CM) and checked by a second (LP), with any discrepancies resolved through discussion.

### 2.4 Meta‐Analysis

Studies were categorised into groups according to intervention and comparator (simulation‐based education vs. patient‐based clinical teaching, simulation‐based education plus clinical teaching vs. clinical teaching only, more vs. less simulation‐based education in course). In each category, meta‐analyses were attempted if there were five or more studies presenting continuous outcomes or presenting categorical outcomes. Meta‐analysis was conducted in Review Manager (RevMan) computer programme (Version 5.4. The Cochrane Collaboration 2020). For each study, if more than one outcome was reported, a single most appropriate outcome was chosen (such as nursing proficiency, final exam results, OSCE results, knowledge test results) before the meta‐analysis was conducted. Normally a higher score indicates a better result. Where the lower score is a better result (such as for number of errors), results were reversed in meta‐analyses. The meta‐analyses were sub‐grouped by study design (RCT, cohort and case control).

Continuous outcomes needed to be presented as means and standard deviations for both groups at follow up. Where studies did not provide SDs but did provide exact *p* values, approximate SDs were calculated (Abbas et al. 2024) and used in meta‐analyses. As studies used a variety of continuous outcome measures, standardised mean differences (SMD) were used in meta‐analyses. SMD is a measure of effect size that has the advantage that it does not depend on the units of measurement used in included studies. However, SMD is reported in units of standard deviation rather than a specific measurement scale so can be more difficult to interpret. Using Cohen's classification of effect sizes, a large effect size is considered to be 0.8, medium 0.5 and small effect 0.2 (Cohen 1977). Results are given as point estimates of SMD with 95% confidence intervals (95% CI). Heterogeneity was assessed using the *I* ^2^ statistic. Funnel plots were constructed to look for publication bias where there were 10 or more studies in one category.

## 3 Results

### 3.1 Search Results

Electronic searches found 9159 citations. There were 4466 duplicates, and 4526 citations were excluded following title and abstract filtering (Figure S1). Full texts of 166 of 167 studies were obtained and 123 excluded (Table S1). There were 42 studies (from 43 papers) included. No additional studies were found from systematic reviews. Eight studies published in Korean (Ahn and Lee 2021; Eom et al. 2010; Hur and Roh 2013; Joo et al. 2015; Kim and Heo 2017; Kim and Kim 2021; Ko and Choi 2017; Park and Kim 2020) were excluded by a Korean speaker, one in Turkish (Tuzer and Yilmazer 2020) by a Turkish speaker and two in Chinese (Xi et al. 2022; Yong and Ying 2020) by a Chinese speaker. Two included studies were written in foreign languages with an English language abstract, one in Chinese (Cao and Cao 2015) and one in Korean (Lee et al. 2014). The Korean and Chinese speakers helped with the data extraction of these papers where essential information was not in the English language abstract. Most studies were excluded because they had the wrong control group—classroom‐based teaching, no teaching at all or another form of simulation. The funnel plot of SBE versus clinical teaching (Figure S2) suggests that small studies with negative or no difference in outcomes may have been missing. If these had been present, they would have had the effect of reducing effect sizes.

### 3.2 Characteristics of Included Studies

Characteristics of the 42 included studies are shown in Table 1. They mostly came from the USA (*N* = 21), but also South Korea (*N* = 7), Saudi Arabia (*N* = 3), Canada (*N* = 2), China (*N* = 2), and one each from the UK, Ghana, Iran, Jordan, Norway, Oman and Turkey. Study designs included 19 RCTs, two randomised crossover trials, three cohort studies and 18 case‐control studies. Study size varied from 12 students (Radnakrishnan et al. 2007) to 847 students (Hayden et al. 2014). All studies included undergraduate nursing students on a variety of courses such as fundamentals of nursing, medical, surgical, mental health, women's health, maternity nursing, family planning, critical care and paediatric nursing. Courses were described as associate degrees, Baccalaureate Nursing, Bachelor of Nursing, Bachelor of Science in Nursing, Diploma of Nursing, Licensed Vocational Nursing, Prelicensure Nursing and undergraduate nursing degrees. Settings included hospital, clinical, nursing home and nursing school venues. Specific teaching used varied from very short courses such as how to insert contraceptive implants (Dery et al. 2019) to the full length of the nursing course (Curl et al. 2016; Guerrero et al. 2021; Guerrero et al. 2022; Guerrero 2023; Hayden et al. 2014; White and Champion 2021).

**TABLE 1:** Characteristics of included studies.

Study name, year (country)	Participants (number)	Study design	Type of course	Specific teaching	Simulation	Debriefing	Control condition	Evaluation	Comment
Alinier et al. 2006 (UK)	Diploma of Higher Education in Nursing (*n* = 99)	RCT	2nd year of course	Clinical scenarios (not described) plus clinical course	Sim man—not described how used	Debriefing given but not described	Clinical course as usual	OSCEs	
Ataee et al. 2019 (Iran)	Bachelor of nursing students (*n* = 37)	RCT	7th semester of course	Coronary care—CPR, working with electroshock device, heart rhythm monitoring, operating ECG	Mannequins	Not mentioned	Internship programme of the coronary care unit	OSCEs	
Bai et al. 2023 (China)	Final year undergraduate nursing students (*n* = 30)	Case control	On clinical practice rotations in the post anaesthesia care unit (PACU)	Four additional in situ simulations during the second and third weeks of their practice in addition to the routine teaching protocol	Four cases based on real life scenarios (30–45 min each)	Given after each simulation	Routine PACU teaching protocol	Clinical judgement	
Banjo‐Ogunnowo and Chisholm 2022 (USA)	Licensed vocational nurses training to be associate degree nurses (*N* = 32)	Case control	Maternal‐paediatric course	Didactic component four credit (64 contact hours) clinical component 3 credit (96 contact hours). Topics not given	i‐Human virtual patient computer programme (4‐h virtual lectures, two 2‐h virtual lab sessions, and 12 h of virtual simulation each week for 8 weeks)	Not mentioned	Four‐hour classroom lectures, two 2‐h labs, and one 12‐h clinical experience per week for 8 weeks	HESI Maternal‐paediatric Specialty exam and conversion scores, and HESI end of programme (Exit) exam	Before and during Covid‐19 comparison
Cao and Cao 2015 (China)	Nursing undergraduates (*N* = 81)	RCT	Obstetrics and gynaecology	Staged simulated examination practice and mini‐clinical evaluation exercise	Not described	Not mentioned	Clinical teaching as usual	Theory and comprehensive clinical skills assessment	Based on English language abstract
Centrella‐Nigro et al. 2016 (USA)	Prelicensure students in a two‐year nursing programme (*N* = 43)	Case control	Whole course	Nursing skills, inter‐professional working	Medium‐fidelity simulators of adult, child, infant, and birthing mother	Not mentioned	Attended course in the previous year	Basic Knowledge Assessment Tool	
Craig et al. 2021 (USA)	3rd Year Batchelor of Science in Nursing (BSN) students (*N* = 80)	RCT	medical‐surgical nursing course	Medication administration	Three scenarios over 4 weeks. Week 1: low‐fidelity simulation Week 2: high‐fidelity simulation	Week 1. enhanced medication administration debrief session Week 4 debrief for all	Week 1: standard training during a skills lab Week 2 and 3: standard training on clinical unit Week 4 simulation	Medication Safety Knowledge Assessment, Medication Safety Critical Element Checklist	Weeks 3 and 4 same for both groups: clinical rotation then high fidelity simulation
Curl et al. 2016 (USA)	Associate degree nursing students (*n* = 124)	Case control	Obstetrics, paediatrics, mental health and critical care	Throughout course (50% course simulation)	Simulation plus clinical teaching (1 h simulation = 2 h clinical teaching) Using HFS in skills lab	Debriefing (lasting as long or up to twice as long as the HFS), focused on clinical reasoning with reflection on decision‐making	Clinical teaching alone	Medical‐surgical post‐test and overall exit exam, Health Education Systems Inc. (HESI) exams (higher score better)	No significant differences between groups' mean standard scores on medical‐surgical pre‐test,
Dery et al. 2019 (Ghana)	Community health nursing students (*N* = 50)	RCT	Family planning	Training to insert contraceptive implants	Low‐tech PVC pipe, latex foam, cotton, and leather simulator with a semi‐hollow, cylindrical, PVC base to mimic bone and a piece of cloth for firmly securing it to the arm of the patient	Not mentioned	Classroom training then watching midwives insert contraceptive implants on the wards	Performance assessment score by clinical expert observer	Very specific training
Guerrero et al. 2021 (Saudi Arabia)	Nursing interns with GPA 3.5 and above (*n* = 30)	RCT	Women's health, children's health, medical‐surgical, critical care	Throughout course	HFS 1 day per week plus 4 days hands on clinical training using HFS	Group debriefing after each session	5 days hands on clinical training	Clinical placement grades, direct observation of procedural skills, clinical evaluations, case study presentations	
Guerrero et al. 2022 (Saudi Arabia)	Bachelor of Science in Nursing students (*n* = 192)	Case control	Adult health nursing, critical care nursing courses	Throughout course	HFS along with traditional laboratory teaching and clinical training.	GAS debriefing model (described in text)	Attending maternal‐health nursing and child health nursing courses	Midterm and final OSCE results	
Guerrero 2023 (Saudi Arabia)	Nursing students enrolled in a critical care nursing course (*n* = 139)	Cohort	Critical care nursing	Students were exposed to HFS as part of their traditional skills laboratory and clinical training for four consecutive academic years. The yearly increase of simulation exposure percentage was from 10 to 40	The HFS trainings include briefing, simulation scenarios, and debriefing. Simulation activities were guided by the Jeffries Simulation Framework	20–40 min after each scenario, based on GAS debriefing model	No control condition (more vs. less simulation)	Midterm and final written exam, midterm and final OSCE results, and clinical performance	
Hall 2015 (USA)	Senior nursing baccalaureate students (*n* = 279)	Retrospective cohort	Nursing course	Maternal‐newborn module	Three critical care scenarios	Debriefing given but not described	Traditional clinical experience	NCLEX proficiency level categories	
Hansen and Bratt 2017 (USA)	Nursing students (*n* = 71 enrolled, *n* = 48 finished full trial)	Randomised crossover trial	Nursing course	First medical‐surgical practicum course	3 HFS days and 1 medium fidelity virtual simulation on pain management, heart failure, COPD/pneumonia and diabetes mellitus	Sim TRACT model debriefing	Traditional clinical experiences on wards	Creighton Competency Evaluation Instrument (CCEI) Scores	Mid‐trial results used
Harris 2011 (USA)	Junior level baccalaureate nursing students (*n* = 71)	RCT	Paediatric nursing	Paediatric and neonatal nursing care	Human patient simulator—basic care of infants, medication administration, infant HPS, and child HPS	Individual and full group discussion after each simulation	Hospital session—orientation and introduction to the wards and facilities	Comprehensive paediatric examination, clinical grades	Control and intervention groups had different sessions
Hayden et al. 2014 (USA) (plus Ingwerson 2015)	Prelicensure nursing students (*n* = 847 enrolled, *n* = 666 completed)	RCT	Entire 2 year course	Throughout course	Simulation for 50% of course or for 25% of course Using mannequins in simulation labs	Not mentioned	Simulation for 10% or less of course	Knowledge, Clinical competency, New Graduate Nurse Performance survey, NCLEX	
Hwang and Park 2020 (S Korea)	Third‐year nursing students (*n* = 66)	Case control	Women's nursing	Preoperative nursing for high‐risk pregnant women who were scheduled for a caesarean section	Standardised patient scenarios	Reported but not described	Case study then clinical practice for the week	Clinical judgement, nursing performance, communica‐tion skill, problem‐solving ability	
Lee et al. 2014 (S. Korea)	Third‐year baccalaureate nursing students (*N* = 36)	Case control	Paediatric nursing clinical practicum	Post operation nursing care for children	Simulation education integrated with problem‐based learning (150 min)	Not mentioned	Regular clinical practicum in a hospital setting	Clinical competency examination	From English language abstract.
Lee and Son 2023 (S. Korea)	Third‐year nursing students (*n* = 92)	RCT	Maternity nursing	Electronic fetal monitoring (EFM)	Problem‐based learning for EFM nursing (240 min) plus EFM nursing simulation (180 min)	60 min debriefing	Conventional clinical practice	EFM nursing knowledge, clinical judgement	
Luctkar‐Flude et al. 2012 (Canada)	Second‐year undergraduate nursing (*n* = 44)	RCT	Health assessment course	Respiratory assessment	High fidelity simulation or standard patient	Not mentioned	Untrained community volunteers	Respiratory assessment performance scores	
Mancini et al. 2019 (USA)	Associate Degree in Nursing and Bachelor of Science in Nursing students (*n* = 586)	Case control	Four semesters of course	Medical, surgical, paediatric, maternal, critical care, final project simulations	Skills laboratories and simulation laboratories	Not mentioned	Clinical hours on same course	NCLEX scores	
Meyer et al. 2011 (USA)	Junior paediatric nursing students (*n* = 120 started, *n* = 116 finished)	Cohort	Clinical place‐ment (8 weeks)	Emergency clinical skills, acute care, post‐op care, skills	Simulation for 25% of course (varied order) using mannequin and mini‐skills lab	Unclear	Before students had the simulation component	Clinical performance scores	
Olaussen et al. 2022 (Norway)	Batchelor of Nursing (*n* = 116 started, *n* = 88 finished)	RCT	Clinical practice of 224 h plus simulation	Chronic respiratory disease, dementia, heart failure	Simulation scenarios for 10.7% of the course	Facilitated debriefing that lasted at least 90 min.	7‐week practice period of 224 h in nursing homes	Knowledge test	
O'Lynn and Krautscheid 2014 (USA)	Baccalaureate Nursing students (male only) (*n* = 32 started, 24 assessed)	Case control	Beginning of junior year, medical/surgical course	Intimate touch	Mannequins for intimate touch practice (90 min)	Reported but not described	Clinical hours on same course	Panel assessment of clinical performance on apical pulse and perineal hygiene	
Radnakrishnan et al. 2007 (USA)	Senior Batchelor of Science in Nursing (BSN) students completing a second degree (*n* = 12)	RCT	Senior clinical course	Two patients with complex diagnoses, one of whom develops a medical emergency	Two one‐hour practice simulations in a 320‐h internship (0.6% of course)	Routine debriefing with reflective discussion	Clinical course as usual	Clinical Simulation Evaluation Tool (includes safety, basic assessment, focused assessment, interventions, delegation and communication)	Simulation also used in the assess‐ment for all students
Raman et al. 2019 (Oman)	Level 4 undergraduate nursing students (*n* = 80 started, *n* = 74 finished)	Case control	Maternity course	Simulation (SP) plus clinical training	Simulation for 25% of course—normal and high‐risk obstetrics, foetal distress, and cardio‐tocography	Debriefing for 30 to 45 min	135 h clinical training	Knowledge question‐aire, Creighton Competency Evaluation Instrument	
Reaves et al. 2020 (USA)	Batchelor degree (accelerated stream) senior students (*n* = 154)	Case control	Public health nursing	Simulation in a fully furnished, one‐bedroom apartment in a teaching hospital	Example scenarios (paraplegia from spina bifida, head lice) 3 h	Reported but not described	Clinical training as usual without simulation	HESI scores, community health and clinical prevention scores	
Reid et al. 2020 (USA)	Baccalaureate and associate degree nursing students (*n* = 62)	Case control	Maternal newborn clinical course	Postpartum haemorrhage	High fidelity mannequins	For both groups at end of day	Clinical experience of same topic	Lasater Clinical Judgement Rubric	
Roberts et al. 2022 (USA)	Prelicensure baccalaureate of nursing students (*n* = 224)	Cohort	Whole course	Simulated clinical teaching	Avatars to simulate patients	Synchronous online debriefings	Standard course before Covid‐19 pandemic, including low‐ to high fidelity mannequins, standardised participants, and commercial virtual simulations	National Council Licensure Examination (NCLEX)	Pre versus during Covid‐19
Schlairet and Pollock 2010 (USA)	Undergraduate nursing students (*n* = 74 started, *n* = 71 finished)	Randomised crossover trial	Funda‐mentals of nursing	Clinical scenarios	Simulation for 2 weeks, using mannequin and mini‐skills lab	Faculty guided debrief	Before students had the simulation component	Knowledge test	
Sears et al. 2010 (Canada)	Second‐year Bachelor of Science in Nursing students (*n* = 54)	RCT	Medical surgical or maternal child nursing	Medication administration	Simulated scenarios regarding medicine administration	Bedside debrief then follow up whilst students filled their knowledge gaps	Medication administration on clinical wards	Medication errors	
Seo and Eom 2021 (S. Korea)	Senior‐year nursing students (*n* = 45)	Case control	Not described	Gastrointestinal tract bleeding, acute myocardial infarction	Simulation for 2 weeks, using videos (otherwise not described)	Mentioned but not described	Intensive care unit clinical practice	Clinical reasoning, problem solving, self‐efficacy, clinical competency	
Soccio 2017 (USA)	Baccalaureate nursing students (*n* = 48)	RCT	Mental health	PTSD, bipolar disorder, mania hearing voices, psychosis, depression and wrist cutting psychiatric emergency scenarios	Simulation for 25% of clinical hours using trained drama students	Theory‐based debriefing method	Clinical hours only	Knowledge (ATI RN Mental Health Mastery Examination)	
Son 2020 (S Korea)	Third‐year nursing students (*n* = 98 started, *n* = 78 finished)	RCT	Maternity nursing	Height of fundus measurement, abdominal circumference measurement, Leopold's manoeuvres, foetal monitoring, foetal heart sound auscultation via fetoscope and Doppler, nitrazine test, nursing care for uterine contraction and delivery pain, vertex delivery mechanism, Ritgen's manoeuvre, foetal and placental expulsion, umbilical ligation, postpartum uterine contraction, and bleeding risk assessment	Simulation for 1 week using a high‐fidelity simulator	On completion of each scenario	Clinical experience of pregnancy, delivery and postpartum nursing or surgical operations	Learning attitude, meta‐cognition and critical thinking	
Tawalbeh 2020 (Jordan)	Baccalaureate nursing students (*n* = 76)	RCT	Critical care	CVD, respiratory and head injury management	Simulation plus lectures plus clinical training using skills lab	20 min debrief after each 2 h scenario	Lectures plus clinical training	Knowledge	
Terzioglu et al. 2016 (Turkey)	Nursing students (*n* = 60)	RCT	Obstetrics and Gynaecology Nursing	Leopold's manoeuvres, teaching breastfeeding, family planning education, teaching vulvar self‐examination and teaching breast self‐examination	Nursing skills lab (videos and models) vs. standard patient	After each activity for all students	clinical practice	Psychomotor skill Communication skill State anxiety level	
Thomas and Barker 2022 (USA)	traditional and advanced second‐degree undergraduate nursing students (*n* = 395)	Case control	Simulation elective	Wound care, medication administration, urinary catheter insertion, and cardiopulmonary resuscitation	High‐fidelity manequin‐based scenarios, or standardised patients	30–40 min after each session	Traditional clinical teaching	NCLEX pass rates, grade point averages, Assessment Technologies Institute (ATI) comprehensive predictor scores	
White and Champion 2021 (USA)	Undergraduate nursing students (*n* = 640)	Case control	Maternal‐newborn and paediatric courses	Throughout course	Simulation as part of course, used standardised scenarios	Not mentioned	No simulation in course	National benchmark exam scores	
Witt et al. 2018 (USA)	Bachelor of Science in Nursing (*n* = 32)	RCT	Mental health	Examination skills, communication skills, appropriate treatments for conditions	Standardised patients (paid professional actors)	Guided scripted debriefing	28 h of mental health theory and 84 h of clinical experience	National Council Licensure Examination	
Woda et al. 2019 (USA)	Baccalaureate nursing students (*n* = 71)	Case control	Medical‐surgical adult nursing	Conflict resolution, interdisciplinary teamwork, and a multiple‐patient simulation	14 supplementary simulations, descriptions not given	None given	Standard clinical teaching with four simulation sessions	Creighton Competency Evaluation Instrument	
Yu and Kang 2017 (S Korea)	Senior nursing students (*n* = 62)	RCT	Internal medicine, surgery, paediatrics, and emergency nursing	Handover scenarios in hypoglycemic attack, postoperative bleeding and dyspnea, acute bronchiolitis, high fever, and mental status change, and acute myocardial infarction	Role play and team‐based simulation	Not mentioned	Theoretical lectures and exposure to clinical practice	Communication scores	
Yu et al. 2021 (S Korea)	Senior nursing students (*n* = 51)	Case control	Paediatrics	Neonatal infection control—basic care, feeding management and skin care and environmental management	Virtual reality simulation	20 min discussion after scenarios	Clinical practice with no simulation	High‐risk neonatal infection control knowledge	

Abbreviations: ATI, Assessment Technologies Institute; CCEI, Creighton Competency Evaluation Instrument; GAS, gather analyse summarise; h, hour; HESI, Health Education Systems Incorporated; min, minutes; NCLEX, National Council Licensure Examination; OSCE, Objective Structured Clinical Examination; PTSD, post‐traumatic stress disorder; PVC, polyvinyl chloride; RCT, randomised controlled trial; SP, standardised patient.

The quality of the included studies varied. Formal quality assessment results are reported in Table S2 (RCTs), Table S3 (Cohort studies) and Table S4 (case–control studies). For the RCTs, many studies lacked description of randomisation, and there was either no blinding of participants or blinding was not described. Also, none of the studies described whether the simulation teaching of nurses improved patient care. None of the cohort and case–control studies described or accounted for confounding factors. Where whole course pass rates were used as the outcome measure (e.g., in Banjo‐Ogunnowo and Chisholm 2022; Curl et al. 2016; Hayden et al. 2014; Roberts et al. 2022), it is less likely that assessors knew group assignment, whereas where assessment was immediately part of the simulation course, knowledge of group assignment was more likely.

### 3.3 Comparisons of Simulation‐Based Education to Patient‐Based Clinical Teaching for Undergraduate Nurses

There were three main types of comparisons:

- Simulation‐based education versus clinical only—28 studies.
- Simulation‐based education plus clinical versus clinical only—11 studies.
- More simulation‐based education versus less simulation‐based educations in course—three studies.

### 3.4 Comparison Examples

The simulation‐based education versus patient‐based clinical teaching studies tended to investigate a relatively short aspect of the nursing course. In the study by Thomas and Barker (2022), for example, volunteers for the simulation‐based education elective had high‐fidelity mannequin‐based scenarios or teaching with standardised patients on wound care, medication administration, urinary catheter insertion and cardiopulmonary resuscitation whereas the comparator group non‐volunteers had their traditional clinical teaching. Two of these studies were comparing and contrasting students who had attended the nursing course before the Covid‐19 pandemic with those attending during the pandemic, when teaching had to move to simulation only (Banjo‐Ogunnowo and Chisholm 2022; Roberts et al. 2022). An example of simulation plus clinical versus clinical only is Alinier et al. (2006) where students were randomised to simulation‐based education on SimMan using various scenarios plus clinical course or clinical course as usual. An example of more simulation‐based education versus less simulation‐based education during the course was Hayden et al. (2014) where nurses randomised to entire courses that had simulation‐based education for 50% of the course were compared to those with 25% of the course and 10% of the course.

### 3.5 Types of Simulation

Types of simulation included high, medium or low fidelity simulation, virtual reality, computer programmes, avatars, mannequins, standardised patients (paid professional actors or trained patients) or simulated scenarios, and were not specifically described in some of the studies where whole courses were compared. Where described, the intensity of simulation was compared to patient‐based clinical training in a 1:2 ratio, so that students would be given, for example, 1 h of simulation for 2 h of clinical training. Debriefing was given in 29 studies, not given in one, and unclear in 12 studies (see Table 1).

### 3.6 Comparator Teaching

Much less information was available about comparator group teaching. For the most part it was described as clinical course as usual, classroom plus standard clinical training on wards, or patient‐based clinical experience of the same topic. For three of the studies, the comparator group was the group of students who had yet to take part in the simulation teaching (Craig et al. 2021; Meyer et al. 2011; Schlairet and Pollock 2010). In Meyer et al. (2011) only the 2‐week scores were simulation versus clinical teaching as all students in the study rotated through the simulation teaching in 2‐week blocks.

### 3.7 Outcome Assessment

All studies included one or more independent assessments of nursing ability, such as exams, OSCEs, knowledge tests, externally rated performance scores and clinical placement grades. No studies assessed patient‐based clinical outcomes, patient acceptability of nursing care given or staff resource time for teaching of the students. Numerical results from all studies are presented in Table 2. Most results were presented as continuous outcomes (means with or without standard deviations, medians and ranges) but several presented categorical outcomes, usually pass rate percentages (Guerrero et al. 2021; Hayden et al. 2014; Mancini et al. 2019; Olaussen et al. 2022; Soccio 2017; Thomas and Barker 2022). Almost all numerical results were around nursing ability and teaching success.

**TABLE 2:** Numerical results from objectively measured outcomes.

Study	Group definition and numbers	Outcome description	Intervention result	2nd intervention result	Control result	Statistical tests used	Comparative results and *p*
Alinier et al. (2006)	Simulation+ clinical (*n* = 49) versus clinical course (*n* = 50)	OSCE results (mean [SD])	61.7 (7.5)	N/A	56.0 (9.5)	Independent sample *t*‐test	*p* = 0.001
Ataee et al. (2019)	Simulation (*n* = 19) versus clinical care (*n* = 18)	OSCE total score	34.1 (4.1)	N/A	16.6 (3.8)	*T*‐tests	*p* = 0.030
Bai et al. (2023)	Simulation + clinical teaching (*n* = 15) versus traditional teaching (*n* = 15)	The Chinese version of the Lasater Clinical Judgement Rubric (mean [SD])	35.6 (1.0)	N/A	32.7 (1.2)	*T*‐tests	*p* < 0.001
Banjo‐Ogunnowo and Chisholm (2022)	Simulation (*n* = 18) versus traditional teaching (*n* = 14)	Raw score maternal‐paediatric specialty exam (mean [SD])	752.9 (148.9)	N/A	766.0 (145.4)	Independent‐samples *t*‐test	*p* = 0.81
		Conversion score maternal‐paediatric specialty exam (mean [SD])	68.0 (10.6)	N/A	69.7 (11.6)		*p* = 69
		Maternal‐paediatric specialty exam NLN nursing judgement subscale (mean [SD])	750.8 (142.5)	N/A	765.6 (147.1)		*p* = 78
		Maternal‐paediatric specialty exam NLN nursing practice subscale (mean [SD])	764.2 (143.9)	N/A	766.5 (146.2)		*p* = 97
		Maternal‐paediatric specialty exam Clinical judgement, clinical decision making, critical thinking subscale (mean [SD])	752.9 (148.9)	N/A	761.7 (152.1)		*p* = 87
		Raw score HESI Exit Exam (mean [SD])	896.8 (148.5)	N/A	859.1 (105.7)		*p* = 0.43
		Conversion score HESI Exit Exam (mean [SD])	81.1 (11.3)	N/A	78.6 (8.4)		*p* = 0.48
		HESI Exit Exam NLN nursing judgement subscale (mean [SD])	891.9 (141.8)	N/A	863.3 (107.5)		*p* = 0.52
		HESI Exit Exam NLN nursing practice subscale (mean [SD])	901.2 (145.6)	N/A	862.5 (120.1)		*p* = 0.42
		HESI Exit Exam Clinical judgement, clinical decision making, critical thinking subscale (mean [SD])	896.8 (148.5)	N/A	863.2 (106.6)		*p* = 0.48
Cao and Cao (2015) (China)	Simulation (*n* = 40) versus traditional practical teaching (*n* = 41)	Theory examination score	92.2 (1.5)	N/A	78.5 (7.0)	*T*‐test	*p* < 0.0001
Centrella‐Nigro et al. (2016)	Simulation group (*n* = 21) versus previous year (*n* = 22)	Medical–Surgical Basic Knowledge Assessment Tool	68.2 (3.5)	N/A	67.1 (6.6)	*T*‐test	*p* = 0.49
Craig et al. (2021)	Additional simulation (*n* = 35) versus standard teaching (*n* = 45)	High‐fidelity Simulation Performance: Medication Safety Critical Element Checklist Scores at Week 4 (mean [SD])	14.7 (2.9)	N/A	12.0 (3.1)	Independent‐samples *t*‐test	*p* < 0.001
		Medication Safety Knowledge Assessment Scores at week 4 (mean [SD])	18.5 (1.9)	N/A	17.8 (2.0)		*p* = NR
		Weeks 4–Week 1 difference (mean [SD])	1.5 (2.2)	N/A	0.6 (2.0)		*p* = 0.075
Curl et al. (2016)	Simulation plus clinical teaching (*n* = 59) versus clinical teaching alone (*n* = 65)	Post Medical‐Surgical Standard Score (mean [SD])	931.0 (NR)	NA	884.0 (NR)	ANOVA (not described)	*p* = 0.08
		Post Medical‐Surgical Conversion Score (mean [SD])	85.1 (NR)	NA	81.1 (NR)		*p* = 0.05
		Exit Exam Standard Score (mean [SD])	936.5 (NR)	NA	885.6 (NR)		*p* = 0.01*
		Exit Exam Conversion Score (mean [SD])	87.0 (NR)	NA	82.6 (NR)		*p* = 0.01
		HESI Clinical Specialty Exam Obstetrics (mean [SD])	837.4 (NR)	NA	801.0 (NR)		*p* = 0.35
		HESI Clinical Specialty Exam Paediatrics (mean [SD])	873.7 (NR)	NA	823.4 (NR)		*p* = 0.08
		HESI Clinical Specialty Exam Mental Health (mean [SD])	850.4 (NR)	NA	802.7 (NR)		*p* = 0.12
Dery et al. (2019)	Simulation (*n* = 25) versus clinical teaching (*n* = 25)	Accuracy of insertion, (*n* [%])	119 (95.2)	NA	98 (78.4)	*T*‐tests	*p* < 0.001
		Insertion time, (mean [SD])	33.6 (1.2)	NA	42.2 (2.0)		*p* < 0.001
		Number of errors committed, (mean [SD])	1.9 (0.1)	NA	2.5 (0.2)		*p* = 0.005
Guerrero et al. (2021)	HFS 1 day per week plus hands on clinical training (*n* = 15) versus hands on clinical training only (*n* = 15)	Women's health nursing (mean [SD])	93.8 (3.4)	NA	79.6 (10.1)	*T*‐tests	*p* < 0.01
		Child health nursing (mean [SD])	91.5 (6.1)	NA	83.1 (9.9)		*p* < 0.01
		Medical‐surgical nursing (mean [SD])	94.7 (3.9)	NA	79.5 (12.6)		*p* < 0.01
		Critical care nursing (mean [SD])	97.4 (1.7)	NA	80.8 (9.9)		*p* < 0.01
		General average of clinical placement grades (mean [SD])	94.6 (2.9)	NA	80.7 (8.0)		*p* < 0.01
		Final written exam (mean [SD])	78.0 (9.1)	NA	92.0 (16.1)		*p* < 0.01
		Final internship grades (mean [SD])	91.2 (3.8)	NA	82.9 (7.4)		*p* < 0.01
		Grade categories	Excellent—11 Very Good—4	NA	Excellent—3 Very Good—6 Good—6	NA	
		Marking categories	A+—3 A—8 B+—4	NA	A+—1 A—2 B+—4 B—2 C+—4 C—2	NA	
Guerrero et al. (2022)	Courses with HFS (*n* = 91) versus those without (*n* = 101)	Final OSCE results (mean [SD]) Adult health nursing	90.92 (7.8)	NA	NA	NR	
		Child health nursing	NA	NA	83.5 (8.7)		
		Critical care nursing	98.0 (1.8)	NA	NA	NR	
		Maternal health nursing	NA	NA	83.7 (7.5)		
		Overall mean (SD) midterm and final OSCE results	92.6 (8.4)		82.7 (7.7)		*p* < 0.01
Guerrero (2023)	40% simulation (*n* = 35) versus 30% and 20% simulation (*n* = 38, *n*‐34) versus 10% simulation (*n* = 32)	Final term OSCE (mean [SD])	94.3 (4.4)	90.5 (5.4) 87.5 (8.0)	82.8 (8.0)	ANOVA	*p* < 0.001
		Final exam	88 (9.3)	87 (6.9) 83.4 (8.7)	76.7 (11.7)	ANOVA	*p* < 0.001
		Mini CEX	96.7 (2.7)	92.7 (3.9) 89.7 (5.7)	85.2 (5.3)	ANOVA	*p* < 0.001
		Direct Observational Procedural Skills	94.9 (3.6)	91.6 (5.0) 87.8 (6.6)	86.0 (6.0)	ANOVA	*p* < 0.001
Hall (2015)	Simulation (*n* = 132) versus non‐simulation (*n* = 147)	Level 0 (*N*, [%])	0 (0.0)	NA	7 (5.3)	NA	NA
		Level 1 (*N*, [%])	14 (9.5)	NA	44 (33.3)		
		Level 2 (*N*, [%])	126 (85.7)	NA	76 (57.6)		
		Level 3 (*N*, [%])	7 (4.8)	NA	5 (3.8)		
Hansen and Bratt (2017)	Simulation first (*n* = 22) versus clinical first (*n* = 26)	Total score	21.7 (3.1)	NA	20.0 (2.8)	NA	*p* = NR
		Assessment	2.6 (0.9)	NA	2.0 (0.9)		
		Communication	4.7 (0.7)	NA	4.8 (0.7)		
		Clinical judgement	8.7 (0.8)	NA	8.2 (0.9)		
		Patient safety	5.6 (1.0)	NA	5.1 (1.2)		
Harris (2011)	Simulation (*N* = 16) versus clinical (*n* = 55)	Comprehensive paediatric examination	65.3 (6.9)	NA	67.5 (8.4)	*T*‐tests	*p* = 0.19
		Clinical grades	3.7 (0.1)	NA	3.4 (0.3)		*p* = 0.001
Hayden et al. (2014)	50% (*n* = 210) versus 25% (234) versus 10% (216)	First time NCLEX pass rates	87.1%	85.5%	88.4%	MANOVA	*p* = NS
	50% (*n* = 211) versus 25% (221) versus 10% (209)	Knowledge (Assessment Technologies Institute Comprehensive Predictor) (mean [SD])	70.1 (7.1)	69.5 (8.6)	69.1 (8.7)	ANOVA (not described)	*p* = 0.478
	50% (*n* = 154) versus 25% (165) versus 10% (153)	Global Assessment of Clinical Competency and Readiness for Practice (mean [SD])	8.23 (1.1)	7.78 (1.14)	7.83 (1.3)		50% > 25% and 10%, *p* = 0.001
	50% (*n* = 230) versus 25% (268) versus 10% (216)	Fundamentals of Nursing Final CCEI Clinical Ratings (mean [SD])	93.9 (14.4)	97.1 (6.2)	96.4 (7.6)		25% + 10% > 50%, *p* = 0.001
	50% (*n* = 231) versus 25% (251) versus 10% (210)	Medical = surgical Nursing Final CCEI Clinical Ratings (mean [SD])	97.9 (7.7)	97.5 (5.6)	96.5 (8.9)		*p* = NS
	50% (*n* = 167) versus 25% (181) versus 10% (180)	Advanced Medical = surgical Nursing Final CCEI Clinical Ratings (mean [SD])	98.4 (5.1)	99.0 (3.2)	97.6 (5.9)		25% > 10%, *p* = 0.025
	50% (*n* = 218) versus 25% (250) versus 10% (225)	Maternal‐newborn Nursing Final CCEI Clinical Ratings (mean [SD])	96.3 (8.6)	96.4 (9.2)	98.2 (5.7)		10% > 25% + 50%, *p* = 0.022
	50% (*n* = 210) versus 25% (248) versus 10% (228)	Paediatric Nursing Final CCEI Clinical Ratings (mean [SD])	95.2 (10.9)	97.8 (6.7)	97.5 (6.2)		25% + 10% > 50%, *p* = 0.001
	50% (*n* = 225) versus 25% (220) versus 10% (220)	Mental Health Nursing Final CCEI Clinical Ratings (mean [SD])	95.8 (12.5)	95.1 (15.8)	97.9 (7.6)		10% > 25% *p* = 0.05
	50% (*n* = 67) versus 25% (90) versus 10% (95)	Community Health Nursing Final CCEI Clinical Ratings (mean [SD])	97.6 (7.1)	99.7 (1.9)	96.9 (7.6)		25% > 10%, *p* = 0.008
	50% (*n* = 286) versus 25% (293) versus 10% (268)	Rate of withdrawal (percentages)	19.2%	11.9%	9.3%	Not described	*p* = 0.002
		Dropped out because no longer wished to participate in study (numbers)	*N* = 31	*N* = 21	*N* = 7		*p* = NR
Hwang and Park (2020)	Simulation (*n* = 34) versus clinical practice (*n* = 32)	Clinical judgement (mean [SD])	3.0 (0.5)	N/A	2.2 (0.5)	ANCOVA	*p* = < 0.001
		Nursing performance (mean [SD])	2.5 (0.2)	N/A	2.0 (0.3)		*p* = < 0.001
		Communication skill (mean [SD])	3.9 (0.9)	N/A	2.9 (0.9)		*p* = < 0.001
		Problem‐solving ability (mean [SD])	2.8 (0.7)	N/A	2.6 (0.5)		*p* = 0.057
Lee et al. (2014)	Simulation (*n* = 17) versus clinical practice (*n* = 18)	Clinical Examination Scores (mean [SD])	59.6 (11.2)	N/A	41.6 (15.6)	N/A	*p* = NR
Lee and Son 2023	Simulation (*n* = 46) versus clinical practice (*n* = 46)	EFM nursing knowledge (mean [SD])	14.2 (2.5)	N/A	14.6 (2.0)	ANCOVA, with GPA as covariant	*p* = 0.37
Luctkar‐Flude et al. (2012)	HFS (*n* = 14) versus SP (*n* = 14) versus community volunteer (*N* = 16)	Respiratory assessment performance scores (mean [SD])	32.9 (4.2)	27.4 (4.9)	28.9 (4.5)	MANOVA?	*p* < 0.01
Mancini et al. (2019)	Simulation cohort (*n* = 315) versus clinical cohort (*n* = 271)	Final NCLEX pass rates (percentages)	84.4%	N/A	88.6%	Not stated	*p* = 0.15
Meyer et al. (2011)	Been to simulation in first 2/4/6 weeks (*n* = 28) versus not yet been to simulation (*n* = 89, 60, 29)	2 weeks performance scores (mean [SD])	24.3 (3.8) (28 observations)	N/A	23.1 (4.0) (89 observations)	Compound Symmetry covariance model with SAS Mixed procedure, repeated measure analysis	*p* = 0.19
		4 weeks performance scores (mean [SD])	26.3 (4.0) (28 observations)	N/A	24.3 (3.8) (60 observations)		*p* = 0.03
		6 weeks performance scores (mean [SD])	26.3 (3.7) (28 observations)	N/A	27.3 (3.2) (29 observations)		*p* = 0.36
Olaussen et al. (2022)	Simulation plus clinical (*n* = 50) versus clinical only (*n* = 38)	Knowledge test (mean [SD])	17.6 (3.6)	N/A	14.0 (4.1)	Independent sample *t*‐tests	*p* < 0.01
		Dropout rate	20.8%	N/A	3.8%	N/A	*p* = NR
O'Lynn and Krautscheid (2014)	Simulation (*n* = 17) versus clinical (*n* = 7)	Student asks permission prior to touch—Apical pulse	4.6 (0.8)	N/A	4.6 (0.5)	Independent sample *t*‐tests	*p* = NS
		Student informs client where touch will occur—Apical pulse	4.5 (0.7)	N/A	4.5 (0.9)		*p* = NS
		Student explains what client might feel—Apical pulse	3.4 (1.1)	N/A	3.5 (1.1)		*p* = NS
		Student offers choice to have assistant present—Apical pulse	1.7 (0.7)	N/A	1.6 (0.5)		*p* = NS
		Student speaks calmly—Apical pulse	4.4 (1.0)	N/A	4.4 (0.5)		*p* = NS
		Student does not show anxiety—Apical pulse	4.4 (0.9)	N/A	4.4 (0.5)		*p* = NS
		Student provides privacy—Apical pulse	4.2 (0.9)	N/A	2.2 (1.1)		*p* < 0.001
		Student minimises exposure of client's body—Apical pulse	4.6 (0.8)	N/A	4.3 (0.6)		*p* = NS
		Student engages client in conversation—Apical pulse	3.6 (1.1)	N/A	3.2 (1.0)		*p* = NS
		Student demonstrates respect for client dignity—Apical pulse	4.6 (0.5)	N/A	4.4 (0.5)		*p* = NS
		Student provides comfort to client—Apical pulse	4.4 (0.8)	N/A	4.1 (0.7)		*p* = NS
		Student asks permission prior to touch—Perineal hygiene	4.1 (1.0)	N/A	3.4 (0.9)	Independent sample *t*‐tests	*p* < 0.01
		Student informs client where touch will occur—Perineal hygiene	3.9 (1.2)	N/A	3.0 (1.1)		*p* < 0.05
		Student explains what client might feel—Perineal hygiene	3.2 (1.2)	N/A	2.9 (1.2)		*p* = NS
		Student offers choice to have assistant present—Perineal hygiene	1.9 (1.1)	N/A	1.5 (0.5)		*p* = NS
		Student speaks calmly—Perineal hygiene	4.2 (1.0)	N/A	3.7 (0.7)		*p* = NS
		Student does not show anxiety—Perineal hygiene	4.0 (1.2)	N/A	3.6 (0.9)		*p* = NS
		Student provides privacy—Perineal hygiene	4.1 (0.9)	N/A	3.1 (1.2)		*p* < 0.01
		Student minimises exposure of client's body—Perineal hygiene	4.2 (1.0)	N/A	3.4 (1.2)		*p* < 0.05
		Student engages client in conversation—Perineal hygiene	3.9 (1.2)	N/A	3.1 (1.3)		*p* < 0.05
		Student demonstrates respect for client dignity—Perineal hygiene	4.4 (0.7)	N/A	3.6 (0.8)		*p* < 0.001
		Student provides comfort to client—Perineal hygiene	4.4 (0.6)	N/A	3.6 (0.8)		*p* < 0.001
Radnakrishnan et al. (2007)	Simulation plus clinical (*n* = 6) versus clinical only (*n* = 6)	Clinical Simulation Evaluation Tool (mean [SD])	34.8 (NR)	N/A	32.2 (NR)	N/A	*p* = NS
Raman et al. (2019)	Simulation plus clinical (*n* = 34) versus clinical (*n* = 40)	Knowledge (mean [SD])	21.1 (4.1)	N/A	20.2 (3.4)	ANCOVA	*p* = 0.306
		Creighton Competency Evaluation Instrument—competency (mean [SD])	19.8 (2.3)	N/A	19.6 (3.2)		*p* = 0.683
Reaves et al. (2020)	Simulation plus clinical (*n* = 79) versus clinical (*n* = 75)	Total mean HESI score (mean [SD])	936 (NR)	N/A	885 (NR)	Independent samples *t*‐test	*p* = 0.02*
		Mean HESI conversion score (mean [SD])	85.4 (NR)	N/A	81.3 (NR)		*p* = NR
		Community health mean score (mean [SD])	931 (NR)	N/A	883 (NR)		*p* = NR
		Clinical prevention and Population health mean score (mean [SD])	931 (NR)	N/A	880 (NR)		*p* = NR
Reid et al. (2020)	Simulation (*n* = 27) versus clinical (*n* = 35)	Lasater Clinical Judgement Rubric (mean [SD])	32.0 (5.4)	N/A	30.3 (6.7)	Independent samples *t*‐test	*p* = 0.295
Roberts et al. (2022)	Covid‐19 group (*n* = 112) versus pre‐covid group (*n* = 112)	NCLEX exit exam scores (mean [SD])	944.3 (116.5)	N/A	956.9 (99.4)	Independent samples *t*‐test	*p* = 0.387
Schlairet and Pollock (2010)	Been to simulation (*N*=NR) versus not yet been to simulation (*N* = NR)	Knowledge test (time 1 only) (mean [SD])	62.9 (NR)	N/A	62.4 (NR)	*T*‐tests	*p* = NS
Sears et al. (2010)	Simulation group (*n* = 24) versus clinical (*n* = 30)	Medication errors in course assessment (number)	7	N/A	24	Chi‐squared test	*p* < 0.001
Seo and Eom (2021)	Simulation (*n* = 25) versus clinical practice (*n* = 20)	Clinical reasoning (mean [SD])	32.0 (16.5)	N/A	18.3 (12.9)	ANCOVA controlling for covariates (not described)	*p* = NR
		Problem solving (mean [SD])	99.9 (11.4)	N/A	76.7 (17.5)		*p* = NR
		Self‐efficacy (mean [SD])	137.4 (22.2)	N/A	112.5 (27.2)		*p* = NR
		Clinical competency (mean [SD])	31.9 (4.5)	N/A	18.9 (7.2)		*p* = NR
Soccio (2017)	Simulation (*n* = 24) versus no simulation (*n* = 24)	ATI test passes (score ≥ 80) (percentages)	67%	N/A	50%	N/A	*p* = NR
		ATI test mean scores	NR	N/A	NR	Dependent *t*‐test	*p* = 0.590
Son (2020)	Simulation (*n* = 47) versus clinical practice (*n* = 31)	Learning attitude (mean [SD])	63.4 (6.6)	N/A	62.2 (6.0)	*t*‐tests	*p* = 0.319
		Metacognition (mean [SD])	48.2 (5.9)	N/A	48.7 (5.4)		*p* = 0.140
		Critical thinking (mean [SD])	106.3 (10.5)	N/A	104.7 (11.4)		*p* = 0.798
Tawalbeh (2020)	Simulation (*n* = 38) versus no simulation (*n* = 38)	Knowledge (mean [SD])	29.8 (4.3)	N/A	23.8 (2.2)	*T*‐test	*p* < 0.001
Terzioglu et al. (2016)	Nursing skills lab (*n* = 20), standardised patient lab (*n* = 19) clinical practice (*n* = 20)	Psychomotor skill (median [range])	73.1 (21–98)	81.5 (45–99)	88.6 (46–100)	Kruskal–Wallis variance analysis	*p* = 0.001
		Communication skill (median [range])	64.9 (32–86)	71.6 (4–97)	79.0 (16–100)		*p* = 0.001
		State anxiety level (median [range])	33.0 (21–67)	32.0 (20–73)	31.0 (20–69)		*p* = 0.418
Thomas and Barker (2022)	Simulation elective (*n* = 102) versus not (*n* = 293)	Assessment Technologies Institute (ATI) comprehensive predictor scores (mean [SD])	74.6 (5.5)	N/A	72.2 (6.9)	NR	*p* = 0.001
		Grade point averages, (mean [SD])	3.55 (0.22)	N/A	3.53 (0.23)		*p* = 0.551
		NCLEX pass rates, (percentages)	94%	N/A	88%	NR	*p* = 0.16
White and Champion (2021)	Simulation (*n* = 358) versus no simulation (*n* = 282)	Benchmark exam scores for maternal‐newborn course (mean [SD])	76.3 (8.0)	N/A	70.2 (6.9)	*T*‐test	*p* < 0.0001
		Benchmark exam scores for paediatric course (mean [SD])	76.8 (7.3)	N/A	72.4 (7.4)		*p* < 0.0001
Witt et al. (2018)	Simulation (*n* = 17) versus no simulation (*n* = 15)	Final examination scores (mean [SD])	87.9 (7.6)	N/A	85.8 (5.7)	Wilcoxon signed rank test	*p* = 0.30
		Kaplan Mental Integrated examination rank (mean [SD])	66.2 (22.3)	N/A	78.0 (19.3)		*p* = 0.12
Woda et al. (2019)	More simulation (*n* = 36) versus less simulation (*n* = 35)	Creighton Competency Evaluation Instrument total score (mean [SD])	14.17 (NR)	N/A	12.44 (NR)	NR	*p* = NR
		Creighton Competency Evaluation Instrument Assessment subscale (mean [SD])	2.28 (NR)	N/A	1.9 (NR)	NR	*p* = NR
Yu and Kang (2017)	Simulation (*n* = 31) versus no simulation (*n* = 31)	Overall SBAR communication scores (mean [SD])	17.3 (3.3)	N/A	14.7 (3.4)	Independent *t*‐tests	*p* = 0.003
		Overall communication clarity scores (mean [SD])	28.7 (3.5)	N/A	23.7 (3.5)		*p* < 0.001
		Handover confidence score (mean [SD])	5.4 (1.5)	N/A	4.5 (1.8)		*p* = 0.054
Yu et al. (2021)	Simulation (*n* = 25) versus no simulation (*n* = 25)	High‐risk neonatal infection control knowledge (mean [SD])	22.8 (2.3)	N/A	22.1 (3.3)	Independent *t*‐test	*p* = 0.288

Abbreviations: ATI, Assessment Technologies Institute; CCEI, Creighton Competency Evaluation Instrument; GAS, gather analyse summarise; h, hour; HESI, Health Education Systems Incorporated; min, minutes; Mini CEX, Mini Clinical Evaluation Exercise; N/A, not applicable; NCLEX, National Council Licensure Examination; NR, not reported; NS, not statistically significant; OSCE, Objective Structured Clinical Examination; PTSD, post‐traumatic stress disorder; PVC, polyvinyl chloride; RCT, randomised controlled trial; SBAR, situation, background, assessment, recommendation; SD, standard deviation; SP, standardised patient.

* SD calculated from *p* values.

### 3.8 Dropout Rates

Two studies also reported dropout rates for either more versus less simulation‐based education (Hayden et al. 2014) or simulation‐based education plus clinical teaching versus clinical teaching only (Olaussen et al. 2022). Both showed higher rates of student dropout with more simulation‐based education, but Olaussen et al. (2022) did not explore reasons. Hayden et al. (2014) suggested that some dropouts might be because of people failing exams, but there was no statistically significant difference in failure rates between the three groups. They also found that there were higher numbers of withdrawals because people no longer wished to take part in the study in the 50% group (*n* = 31) and the 25% group (*n* = 21) compared to the 10% group (*n* = 7).

### 3.9 Staff Training

Regarding training staff who delivered the simulation, 31 included studies gave no details. Of these, some studies may have had a single simulation trainer who was also the author of the paper, but this was not clear. In the remaining 11 studies, only two explained how the staff delivering the simulation were trained. Meyer et al. (2011) described how simulation‐trained master's degree teaching assistants were paired with inexperienced facilitators, whereas Raman et al. (2019) described how all simulation facilitators were trained by a simulation expert certified by the Society for Simulation in Healthcare (SSIH).

### 3.10 Outcomes and Meta‐Analysis Results

Three studies compared more versus less simulation‐based education in whole courses (Guerrero 2023; Hayden et al. 2014; Woda et al. 2019). Two demonstrated similar results for each group, such as National Council Licensure Examination (NCLEX) pass rates (Hayden et al. 2014) and Creighton Competency Evaluation Instrument total scores (Woda et al. 2019), but Guerrero (2023) found improved results for those given more simulation‐based education.

**FIGURE 1:**
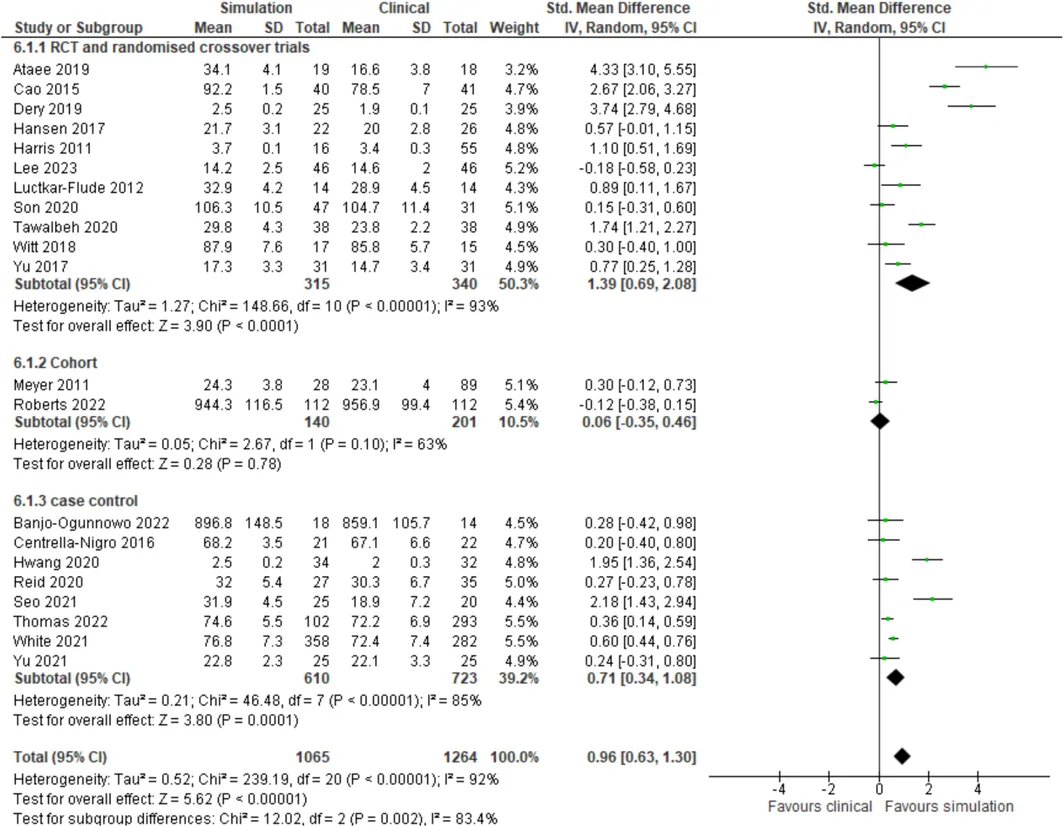
Meta‐analysis of simulation‐based education versus clinical teaching. CI, confidence interval; df, degrees of freedom; IV, inverse variance; RCT, randomised controlled trial; SD, standard deviation; Std, standardised.

Twenty‐eight studies reported simulation‐based education versus clinical teaching, and 21 were used for the meta‐analysis (see Table 3). Seven studies could not be included for specific reasons. Four gave categorical outcomes only. Hall (2015) gave the outcome of proficiency as percentages, Mancini et al. (2019) gave final NCLEX pass rates as percentages, Sears et al. (2010) gave the numbers of medication errors and Soccio (2017) gave the number of ATI test passes. O'Lynn and Krautscheid (2014) gave results of very specific evaluation panel assessment questions with no overall summation. In Schlairet and Pollock (2010), the eligible outcome was a knowledge test, but the size of each group was not given, and in Terzioglu et al. (2016), psychomotor skill results were given as medians and ranges rather than means and standard deviations. The meta‐analysis is shown in Figure 1. In all 21 studies, simulation‐based education was as effective or more effective than clinical teaching only. The SMD was 0.96 (95% CI: 0.63 to 1.30). So, on average, simulation‐based education was more effective than patient‐based clinical teaching in improving the mix of outcomes measured. The effect size was apparently large at 0.96, but heterogeneity was 92%.

**TABLE 3:** Studies and outcomes used for the two meta‐analyses.

Study	Outcomes used
**Simulation‐based education vs clinical teaching**	
Ataee et al. (2019)	Objective Structured Clinical Examination total score
Banjo‐Ogunnowo and Chisholm (2022)	Raw Health Educations System Incorporated (HESI exit exam results)
Cao and Cao (2015)	Theory examination score
Centrella‐Nigro et al. (2016)	Basic knowledge assessment
Dery et al. (2019)	Mean number of errors committed (reversed)
Hansen and Bratt (2017)	Total score
Harris (2011)	Clinical grades
Hwang and Park (2020)	Nursing performance
Lee and Son (2023)	Knowledge test
Luctkar‐Flude et al. (2012)	Respiratory assessment performance scores
Meyer et al. (2011)	2‐week performance scores
Reid et al. (2020)	Lasater Clinical Judgement Rubric
Roberts et al. (2022)	National Council Licensure Examination exit exam scores
Seo and Eom (2021)	Clinical competency
Son (2020)	Critical thinking
Tawalbeh (2020)	Knowledge
Thomas and Barker (2022)	Assessment Technologies Institute (ATI comprehensive predictor scores)
White and Champion (2021)	Benchmark exam scores for paediatric course
Witt et al. (2018)	Final examination scores
Yu and Kang (2017)	Overall Situation, Background, Assessment, Recommendation (SBAR communication scores)
Yu et al. (2021)	High‐risk neonatal infection control knowledge
**Simulation‐based education plus clinical teaching vs clinical teaching only**	
Alinier et al. (2006)	Objective Structured Clinical Examination results
Bai et al. (2023)	Lasater Clinical Judgement
Craig et al. (2021)	Medication Safety Knowledge Assessment Scores at week 4
Curl et al. (2016)	Exit Exam Standard Score
Guerrero et al. (2021)	Final internship grades
Guerrero et al. (2022)	Final Objective Structured Clinical Examination results
Lee et al. (2014)	Mean Clinical Examination Scores
Olaussen et al. (2022)	Knowledge test
Radnakrishnan et al. (2007)	Clinical Simulation Evaluation Tool
Raman et al. (2019)	Creighton Competency Evaluation Instrument—competency
Reaves et al. (2020)	Total mean Health Educations System Incorporated (HESI) score

**FIGURE 2:**
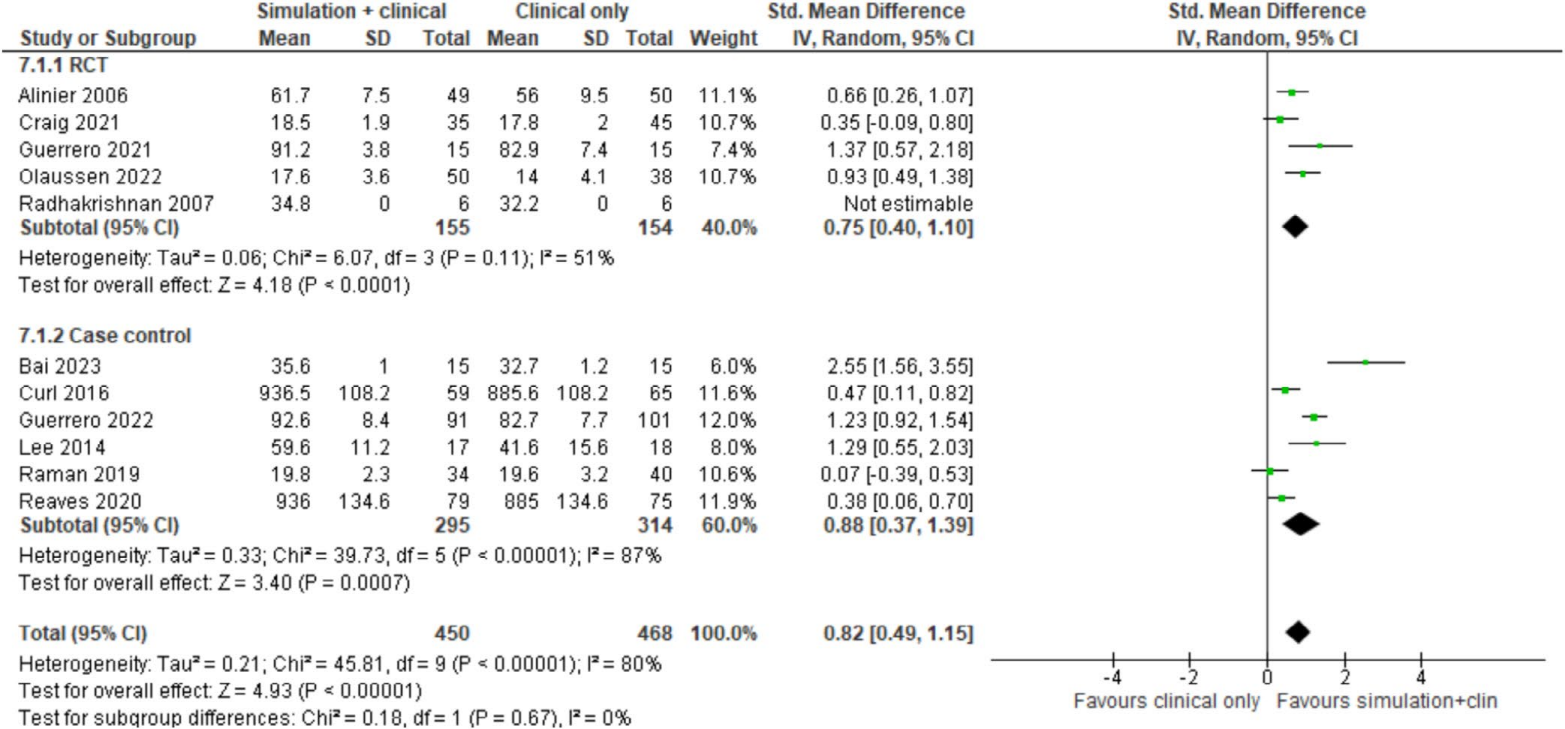
Meta‐analysis of simulation‐based education plus clinical teaching versus clinical teaching only. CI, confidence interval; df, degrees of freedom; IV, inverse variance; SD, standard deviation; Std, standardised.

Eleven studies reported simulation‐based education plus clinical teaching versus clinical teaching only (see Table 3). The meta‐analysis is shown in Figure 2. Three of the studies did not contribute to the meta‐analysis result (Curl et al. 2016; Radnakrishnan et al. 2007; Reaves et al. 2020) as they did not report standard deviations. Average SDs were calculated for Curl et al. (2016) and Reaves et al. (2020) as they had precise *p* values for the comparisons. In all studies in the meta‐analysis, simulation‐based education plus patient‐based clinical teaching was as effective or more effective than patient‐based clinical teaching only. The SMD was 0.82 (95% CI: 0.49 to 1.15). So on average, simulation‐based education plus patient‐based clinical teaching was apparently more effective than patient‐based clinical teaching only, with a large effect size, but heterogeneity was 80%.

The evidence included in these meta‐analyses is subject to a number of limitations. Firstly, because RCTs, cohort studies and case–control studies are included, the quality of the body of evidence is low. There is a relatively high likelihood of bias (see Tables S2–S4) as many of the quality assessment items assessed were not present, missing or unclear. Both of the Forest plots showed heterogeneity, but the effect sizes were large. There is also a relatively high probability of publication bias from the funnel plot (see Figure S2). As a result of these factors, it is suggested that the outcomes as a whole have a very low‐quality rating.

## 4 Discussion

### 4.1 Summary of Results

This is the first systematic review that meta‐analyses results from studies comparing simulation‐based education to patient‐based clinical teaching in undergraduate nurses, using objectively assessed outcomes. The 42 included studies provide evidence that simulation‐based education may be used to substitute some patient‐based clinical teaching on the same topic without reduction in course pass rates, competency or knowledge tests. The two meta‐analysis results apparently showed large effect sizes, and none of the included studies had worse outcomes with simulation‐based education. However, these effect sizes should be interpreted cautiously given high heterogeneity, risk of bias and probable publication bias.

Two of the studies evaluating more versus less simulation‐based education in the curriculum (Hayden et al. 2014; Woda et al. 2019) showed little difference in outcomes, and the study with the largest participant numbers (Hayden et al. 2014) showed little difference in course pass rates between 50% simulation, 25% simulation and 10% simulation. Only one smaller study (Guerrero 2023) showed consistently higher course pass rates with higher amounts of simulation‐based education in the course. There was no evidence on patient‐based outcomes (Kirkpatrick Level 4) or resource implications, but weak evidence from two studies suggested higher drop‐out rates with more simulation‐based education (Hayden et al. 2014; Olaussen et al. 2022). Precise details on the simulation teaching used were missing from some studies, such as duration of scenarios, time allocated, the nature, quality and extent of debriefing used. Few described carefully the educational contents in order to fully understand the interventions. Most of the studies did not report whether the simulation facilitators were appropriately qualified or trained to facilitate simulation activities and deliver effective debriefing.

Some evidence was from simulated learning introduced as a result of the Covid‐19 pandemic. Because of other difficulties that were happening to people during as opposed to before the pandemic, historical controls may not be that comparable. However, in spite of this, the cohorts during the pandemic did just as well as those before the pandemic.

### 4.2 Strengths and Limitations

A major strength of this systematic review is the precise educational question. A difficulty during its conduct was the large number of publications evaluating simulation‐based education in nursing, which meant sifting through much evidence in order to find relevant studies. Much of the excluded evidence compared simulation‐based education to classroom‐based teaching. Studies were excluded where they were not clear about the location of teaching received by the comparator group.

Another strength was the use of already published systematic reviews to find includable studies. It was useful to note that some of the studies found in the database searches had already been found through the systematic reviews but that none of the included studies were only found through searching the systematic reviews. Search terms used in different databases were a trade‐off between sensitivity and specificity so there may be evidence not found; but given the high number of included studies, it would be unlikely that any missing evidence would alter the findings considerably.

An implicit assumption in the way this systematic review and meta‐analyses have been conducted is that different forms of simulation‐based education are equivalent. However, it is acknowledged that this is far from true. Simulation‐based education can involve using real people such as standardised patients, or mannequins in real beds, or virtual reality and other computerised scenarios. Some of the included studies used several forms. There was insufficient information from included studies to establish whether there were consistent differences in educational assessments comparing, for example, real life simulation to virtual reality. In addition to this, the outcome measures used in the studies were varied and are not equivalent. These two factors make it challenging to compare findings across studies.

The meta‐analyses also could be seen to assume that different outcome measures are equivalent. However, in a meta‐analysis it is the comparison between groups that is important, so only studies that used the same outcome measure for intervention and control groups were included. It is this comparison that is meta‐analysed so absolute differences between outcome measures are not as important.

This systematic review could only include studies where the same topic could be taught by simulation‐based education and through clinical or patient‐based teaching in hospitals and other similar environments. Unusual events that nurses need to know about, such as catastrophes, natural disasters and terrorist attacks, are rare, so it is likely that they could only be taught through simulation‐based education. These are not covered by this systematic review.

In an effectiveness question there is a well‐established hierarchy of evidence from the various study designs, starting at RCTs, then cohort studies then case control studies. This information has been presented in Table 1 to give the reader some information on the study design and so the strength of the evidence. Many of the case–control studies could have used random allocation and if this had been done evidence strength would have improved. Because of the very low‐quality grading of the evidence, the magnitude of the meta‐analyses effect sizes is not thought to be accurate, but only give an indication of outcome direction. The meta‐analyses assume that evidence from RCTs, cohort and case–control studies is equivalently robust, and this is not the case. However, if the meta‐analyses were split by study design, there would be too little evidence in each to demonstrate an effect. Therefore, subgroup meta‐analyses by study design have been presented instead.

### 4.3 Implications for Policymakers

The UK Nursing and Midwifery Council (NMC) currently allow 600 practice hours (26% of total teaching hours) to be completed through simulation in approved education institutions (NMC 2022). The findings of this systematic review suggest that, within the contexts studied, substitution of some patient‐based clinical teaching with simulation does not appear to disadvantage students in objectively assessed educational outcomes. However, the substantial heterogeneity, methodological limitations and absence of patient‐level outcome data mean that caution is warranted in extrapolating these findings to broader curriculum redesign.

A recent UK Delphi study that suggested that between 11% and 30% of patient‐based clinical training time could be replaced with simulation‐based education (Bridge et al. 2022). In the US, regulatory bodies have progressively expanded approvals, and SBE is now authorised to substitute for up to 50% of required clinical hours in many States (Smiley and Martin 2023).

Some UK nurse training establishments use no or very little simulation‐based education currently, so there may be a need for training of the trainers to bring them to the current position in training establishments that use more simulation‐based education. This would increase throughput of nurses in training, as one limiting factor is the current lack of clinical patient‐based teaching placements available. In future, virtual hospitals may be used.

The original study protocol listed staff resource time for teaching of the students as an important primary outcome, but no included studies reported this. Decisions regarding increasing the proportion of simulation within nursing programmes should therefore consider not only educational assessment outcomes but also resource requirements, costs, staff training, infrastructure capacity and the impact on patient care environments. Importantly, no included studies evaluated cost‐effectiveness, staff workload, or patient‐level clinical outcomes, all of which are relevant to policy decisions, particularly for resources to be made available to follow where nurses are being trained.

### 4.4 Implications for Research

Simulation‐based education may offer flexibility in contexts where clinical placement capacity is constrained. However, further robust research is required to determine the optimal balance between simulation and patient‐based clinical teaching to ensure maintenance of clinical competence and public safety standards. The evidence‐base remains largely confined to short‐term educational outcomes rather than patient‐level indicators of care quality, safety, or longer‐term professional competence. None of the included studies reported these patient‐based outcomes, yet the main driver for the 2300 clinical practice hours requirement in the UK, for example, is to ensure adequate patient‐based skills in nursing graduates. Consequently, while substitution of some clinical hours with simulation appears unlikely to compromise students' assessment performance, the optimal proportion, design, and implementation of simulation within curricula remain uncertain. Greater standardisation of outcome measures would also strengthen future evidence synthesis.

Future research should prioritise robust comparative designs, transparent reporting, and inclusion of patient and service‐level outcomes. The possibility of higher student dropout rates with more simulation should be investigated, why this might be happening, and whether alterations to the way simulation teaching is delivered might alter this. Research should therefore move beyond the question of whether simulation ‘works’ to examine how, for whom, and under what conditions simulation‐based education is most effective.

Methodological limitations were common across studies, including inadequate reporting of randomisation procedures, lack of control for confounding in non‐randomised designs, and limited transparency regarding comparator clinical exposure. Future evaluations should employ robust comparative designs, clearly define intervention and comparator components, and report sufficient statistical detail to enable synthesis.

### 4.5 Conclusions

Evidence from 42 comparative studies suggests that simulation‐based education is at least comparable to patient‐based clinical teaching for objectively assessed educational outcomes in undergraduate nursing students and may be associated with higher assessment scores in some contexts. However, the magnitude of effect should be interpreted cautiously due to substantial heterogeneity, risk of bias across included studies, and probable publication bias. Importantly, no included studies evaluated patient‐level clinical outcomes, resource implications, or long‐term practice performance. While simulation‐based education appears unlikely to disadvantage students when substituting for some clinical hours, stronger, methodologically robust research is required before firm conclusions can be drawn regarding optimal proportions of simulation within nursing curricula.

## Author Contributions

**Louise Prothero:** searches, citation checking, data extraction, review and editing, validation, conceptualisation. **Naim Abdulmohdi:** writing – original draft, review and editing, validation, formal analysis and synthesis, methodology, conceptualisation. **Siân Shaw:** review and editing, methodology, conceptualisation. **Mary Edmonds:** writing, review and editing, validation, formal analysis, project administration, methodology, conceptualisation. **Catherine Meads:** citation checking, data extraction, review and editing, supervision, conceptualisation.

## Funding

Project commissioned and funded by Health Education England (HEE), now NHS England (NHSE) and Council of Deans of Health (CoDH). The views expressed in this publication are those of the authors and not those of NHSE and CoDH.

## Ethics Statement

The authors have nothing to report.

## Consent

The authors have nothing to report.

## Conflicts of Interest

The authors declare no conflicts of interest.

## Supporting information


**Figure S1:** PRISMA flow diagram.
**Figure S2:** Funnel plot of studies comparing simulation versus no simulation.
**Table S1:** List of excluded studies and reasons for exclusion.
**Table S2:** RCT and randomised crossover trial results.
**Table S3:** Cohort study results.
**Table S4:** Case control study results.

## Data Availability

Data sharing not applicable to this article as no datasets were generated or analysed during the current study.
