# Supplementary material for: The Effectiveness of Simulation‐Based Versus Patient‐Based Clinical Education in Nursing: A Systematic Review and Meta‐Analysis
Source: Nurs Open. 2026 Sep 4;13(9):e70689. doi: 10.1002/nop2.70689 (PMC13545275; doi:10.1002/nop2.70689)
Supplement: Supplementary file 1 [file NOP2-13-e70689-s001.docx]

## Online supplement

Title: **The effectiveness of simulation-based versus patient-based clinical education in nursing; a systematic review and meta-analysis**

**Contents**

Search strategies for Medline, Embase, CINAHL, PsychInfo and Cochrane Central databases

Figure S1. PRISMA flow diagram

Figure S2. Funnel plot

References of included studies

Table S1. List of excluded studies and reasons for exclusion

Quality assessment checklist results

Table S2. RCT results

Table S3. Cohort study results

Table S4. Case control study results

**Medline search strategy**

1.	simulation training/

2.	simulat*.mp. [mp=title, book title, abstract, original title, name of substance word, subject heading word, floating sub-heading word, keyword heading word, organism supplementary concept word, protocol supplementary concept word, rare disease supplementary concept word, unique identifier, synonyms]

3.	proxy for.mp. [mp=title, book title, abstract, original title, name of substance word, subject heading word, floating sub-heading word, keyword heading word, organism supplementary concept word, protocol supplementary concept word, rare disease supplementary concept word, unique identifier, synonyms]

4.	virtual realit*.mp. [mp=title, book title, abstract, original title, name of substance word, subject heading word, floating sub-heading word, keyword heading word, organism supplementary concept word, protocol supplementary concept word, rare disease supplementary concept word, unique identifier, synonyms]

5.	mixed realit*.mp. [mp=title, book title, abstract, original title, name of substance word, subject heading word, floating sub-heading word, keyword heading word, organism supplementary concept word, protocol supplementary concept word, rare disease supplementary concept word, unique identifier, synonyms]

6.	extended realit*.mp. [mp=title, book title, abstract, original title, name of substance word, subject heading word, floating sub-heading word, keyword heading word, organism supplementary concept word, protocol supplementary concept word, rare disease supplementary concept word, unique identifier, synonyms]

7.	XR.mp. [mp=title, book title, abstract, original title, name of substance word, subject heading word, floating sub-heading word, keyword heading word, organism supplementary concept word, protocol supplementary concept word, rare disease supplementary concept word, unique identifier, synonyms]

8.	mannekin*.mp. [mp=title, book title, abstract, original title, name of substance word, subject heading word, floating sub-heading word, keyword heading word, organism supplementary concept word, protocol supplementary concept word, rare disease supplementary concept word, unique identifier, synonyms]

9.	mannequin*.mp. [mp=title, book title, abstract, original title, name of substance word, subject heading word, floating sub-heading word, keyword heading word, organism supplementary concept word, protocol supplementary concept word, rare disease supplementary concept word, unique identifier, synonyms]

10.	avatar*.mp. [mp=title, book title, abstract, original title, name of substance word, subject heading word, floating sub-heading word, keyword heading word, organism supplementary concept word, protocol supplementary concept word, rare disease supplementary concept word, unique identifier, synonyms]

11.	role-play*.mp. [mp=title, book title, abstract, original title, name of substance word, subject heading word, floating sub-heading word, keyword heading word, organism supplementary concept word, protocol supplementary concept word, rare disease supplementary concept word, unique identifier, synonyms]

12.	visualisation*.mp. [mp=title, book title, abstract, original title, name of substance word, subject heading word, floating sub-heading word, keyword heading word, organism supplementary concept word, protocol supplementary concept word, rare disease supplementary concept word, unique identifier, synonyms]

13.	virtual*.mp. [mp=title, book title, abstract, original title, name of substance word, subject heading word, floating sub-heading word, keyword heading word, organism supplementary concept word, protocol supplementary concept word, rare disease supplementary concept word, unique identifier, synonyms]

14.	mock-up*.mp. [mp=title, book title, abstract, original title, name of substance word, subject heading word, floating sub-heading word, keyword heading word, organism supplementary concept word, protocol supplementary concept word, rare disease supplementary concept word, unique identifier, synonyms]

15.	imitat*.mp. [mp=title, book title, abstract, original title, name of substance word, subject heading word, floating sub-heading word, keyword heading word, organism supplementary concept word, protocol supplementary concept word, rare disease supplementary concept word, unique identifier, synonyms]

16.	2 or 3 or 4 or 5 or 6 or 7 or 8 or 9 or 10 or 11 or 12 or 13 or 14 or 15

17.	train*.mp. [mp=title, book title, abstract, original title, name of substance word, subject heading word, floating sub-heading word, keyword heading word, organism supplementary concept word, protocol supplementary concept word, rare disease supplementary concept word, unique identifier, synonyms]

18.	educat*.mp. [mp=title, book title, abstract, original title, name of substance word, subject heading word, floating sub-heading word, keyword heading word, organism supplementary concept word, protocol supplementary concept word, rare disease supplementary concept word, unique identifier, synonyms]

19.	teach*.mp. [mp=title, book title, abstract, original title, name of substance word, subject heading word, floating sub-heading word, keyword heading word, organism supplementary concept word, protocol supplementary concept word, rare disease supplementary concept word, unique identifier, synonyms]

20.	learn*.mp. [mp=title, book title, abstract, original title, name of substance word, subject heading word, floating sub-heading word, keyword heading word, organism supplementary concept word, protocol supplementary concept word, rare disease supplementary concept word, unique identifier, synonyms]

21.	17 or 18 or 19 or 20

22.	16 and 21

23.	1 or 22

24.	nursing/

25.	nurs*.mp. [mp=title, book title, abstract, original title, name of substance word, subject heading word, floating sub-heading word, keyword heading word, organism supplementary concept word, protocol supplementary concept word, rare disease supplementary concept word, unique identifier, synonyms]

26.	24 or 25

27.	23 and 26

28.	exam.mp.

29.	exam*.mp. [mp=title, book title, abstract, original title, name of substance word, subject heading word, floating sub-heading word, keyword heading word, organism supplementary concept word, protocol supplementary concept word, rare disease supplementary concept word, unique identifier, synonyms]

30.	28 or 29

31.	OSCE.mp. [mp=title, book title, abstract, original title, name of substance word, subject heading word, floating sub-heading word, keyword heading word, organism supplementary concept word, protocol supplementary concept word, rare disease supplementary concept word, unique identifier, synonyms]

32.	competenc*.mp. [mp=title, book title, abstract, original title, name of substance word, subject heading word, floating sub-heading word, keyword heading word, organism supplementary concept word, protocol supplementary concept word, rare disease supplementary concept word, unique identifier, synonyms]

33.	30 or 31 or 32

34.	27 and 33

35.	limit 34 to yr="2000 -Current"

**Embase search strategy**

1	simulation training/	8147

2	simulat*.mp. [mp=title, abstract, heading word, drug trade name, original title, device manufacturer, drug manufacturer, device trade name, keyword heading word, floating subheading word, candidate term word]	789756

3	proxy for.mp. [mp=title, abstract, heading word, drug trade name, original title, device manufacturer, drug manufacturer, device trade name, keyword heading word, floating subheading word, candidate term word]	38532

4	virtual realit*.mp. [mp=title, abstract, heading word, drug trade name, original title, device manufacturer, drug manufacturer, device trade name, keyword heading word, floating subheading word, candidate term word]	29905

5	mixed realit*.mp. [mp=title, abstract, heading word, drug trade name, original title, device manufacturer, drug manufacturer, device trade name, keyword heading word, floating subheading word, candidate term word]	845

6	extended realit*.mp. [mp=title, abstract, heading word, drug trade name, original title, device manufacturer, drug manufacturer, device trade name, keyword heading word, floating subheading word, candidate term word]	131

7	XR.mp. [mp=title, abstract, heading word, drug trade name, original title, device manufacturer, drug manufacturer, device trade name, keyword heading word, floating subheading word, candidate term word]	8392

8	mannekin*.mp. [mp=title, abstract, heading word, drug trade name, original title, device manufacturer, drug manufacturer, device trade name, keyword heading word, floating subheading word, candidate term word]	7

9	mannequin*.mp. [mp=title, abstract, heading word, drug trade name, original title, device manufacturer, drug manufacturer, device trade name, keyword heading word, floating subheading word, candidate term word]	3340

10	avatar*.mp. [mp=title, abstract, heading word, drug trade name, original title, device manufacturer, drug manufacturer, device trade name, keyword heading word, floating subheading word, candidate term word]	2510

11	role-play*.mp. [mp=title, abstract, heading word, drug trade name, original title, device manufacturer, drug manufacturer, device trade name, keyword heading word, floating subheading word, candidate term word]	41625

12	visualisation*.mp. [mp=title, abstract, heading word, drug trade name, original title, device manufacturer, drug manufacturer, device trade name, keyword heading word, floating subheading word, candidate term word]	13045

13	virtual*.mp. [mp=title, abstract, heading word, drug trade name, original title, device manufacturer, drug manufacturer, device trade name, keyword heading word, floating subheading word, candidate term word]	204927

14	mock-up*.mp. [mp=title, abstract, heading word, drug trade name, original title, device manufacturer, drug manufacturer, device trade name, keyword heading word, floating subheading word, candidate term word]	687

15	imitat*.mp. [mp=title, abstract, heading word, drug trade name, original title, device manufacturer, drug manufacturer, device trade name, keyword heading word, floating subheading word, candidate term word]	18216

16	2 or 3 or 4 or 5 or 6 or 7 or 8 or 9 or 10 or 11 or 12 or 13 or 14 or 15	1081068

17	train*.mp. [mp=title, abstract, heading word, drug trade name, original title, device manufacturer, drug manufacturer, device trade name, keyword heading word, floating subheading word, candidate term word]	991550

18	educat*.mp. [mp=title, abstract, heading word, drug trade name, original title, device manufacturer, drug manufacturer, device trade name, keyword heading word, floating subheading word, candidate term word]	1530938

19	teach*.mp. [mp=title, abstract, heading word, drug trade name, original title, device manufacturer, drug manufacturer, device trade name, keyword heading word, floating subheading word, candidate term word]	350583

20	learn*.mp. [mp=title, abstract, heading word, drug trade name, original title, device manufacturer, drug manufacturer, device trade name, keyword heading word, floating subheading word, candidate term word]	808450

21	17 or 18 or 19 or 20	2880381

22	16 and 21	147374

23	1 or 22	147374

24	nursing/	205636

25	nurs*.mp. [mp=title, abstract, heading word, drug trade name, original title, device manufacturer, drug manufacturer, device trade name, keyword heading word, floating subheading word, candidate term word]	858671

26	24 or 25	858671

27	23 and 26	15082

28	exam.mp.	86707

29	exam*.mp. [mp=title, abstract, heading word, drug trade name, original title, device manufacturer, drug manufacturer, device trade name, keyword heading word, floating subheading word, candidate term word]	5083238

30	28 or 29	5083238

31	OSCE.mp. [mp=title, abstract, heading word, drug trade name, original title, device manufacturer, drug manufacturer, device trade name, keyword heading word, floating subheading word, candidate term word]	3528

32	competenc*.mp. [mp=title, abstract, heading word, drug trade name, original title, device manufacturer, drug manufacturer, device trade name, keyword heading word, floating subheading word, candidate term word]	232391

33	30 or 31 or 32	5272405

34	27 and 33	5530

35	patient.mp. [mp=title, abstract, heading word, drug trade name, original title, device manufacturer, drug manufacturer, device trade name, keyword heading word, floating subheading word, candidate term word]	6746319

36	hospital.mp. [mp=title, abstract, heading word, drug trade name, original title, device manufacturer, drug manufacturer, device trade name, keyword heading word, floating subheading word, candidate term word]	2622070

37	clinic*.mp. [mp=title, abstract, heading word, drug trade name, original title, device manufacturer, drug manufacturer, device trade name, keyword heading word, floating subheading word, candidate term word]	13339477

38	practice.mp. [mp=title, abstract, heading word, drug trade name, original title, device manufacturer, drug manufacturer, device trade name, keyword heading word, floating subheading word, candidate term word]	1892612

39	35 or 36 or 37 or 38	16760544

40	34 and 39	5075

41	trial*.mp. [mp=title, abstract, heading word, drug trade name, original title, device manufacturer, drug manufacturer, device trade name, keyword heading word, floating subheading word, candidate term word]	2887088

42	control*.mp. [mp=title, abstract, heading word, drug trade name, original title, device manufacturer, drug manufacturer, device trade name, keyword heading word, floating subheading word, candidate term word]	12944324

43	comparator*.mp. [mp=title, abstract, heading word, drug trade name, original title, device manufacturer, drug manufacturer, device trade name, keyword heading word, floating subheading word, candidate term word]	28771

44	study.mp. [mp=title, abstract, heading word, drug trade name, original title, device manufacturer, drug manufacturer, device trade name, keyword heading word, floating subheading word, candidate term word]	19452371

45	test.mp. [mp=title, abstract, heading word, drug trade name, original title, device manufacturer, drug manufacturer, device trade name, keyword heading word, floating subheading word, candidate term word]	3586583

46	random*.mp. [mp=title, abstract, heading word, drug trade name, original title, device manufacturer, drug manufacturer, device trade name, keyword heading word, floating subheading word, candidate term word]	2110487

47	experiment*.mp. [mp=title, abstract, heading word, drug trade name, original title, device manufacturer, drug manufacturer, device trade name, keyword heading word, floating subheading word, candidate term word]	5548236

48	effect*.mp. [mp=title, abstract, heading word, drug trade name, original title, device manufacturer, drug manufacturer, device trade name, keyword heading word, floating subheading word, candidate term word]	11092743

49	comparison.mp. [mp=title, abstract, heading word, drug trade name, original title, device manufacturer, drug manufacturer, device trade name, keyword heading word, floating subheading word, candidate term word]	2526783

50	41 or 42 or 43 or 44 or 45 or 46 or 47 or 48 or 49	26597914

51	40 and 50	3890

52	student*.mp. [mp=title, abstract, heading word, drug trade name, original title, device manufacturer, drug manufacturer, device trade name, keyword heading word, floating subheading word, candidate term word]	582931

53	pre-registration.mp. [mp=title, abstract, heading word, drug trade name, original title, device manufacturer, drug manufacturer, device trade name, keyword heading word, floating subheading word, candidate term word]	2317

54	pre-licensure.mp. [mp=title, abstract, heading word, drug trade name, original title, device manufacturer, drug manufacturer, device trade name, keyword heading word, floating subheading word, candidate term word]	448

55	baccalaureate.mp. [mp=title, abstract, heading word, drug trade name, original title, device manufacturer, drug manufacturer, device trade name, keyword heading word, floating subheading word, candidate term word]	5000

56	undergraduate.mp. [mp=title, abstract, heading word, drug trade name, original title, device manufacturer, drug manufacturer, device trade name, keyword heading word, floating subheading word, candidate term word]	54443

57	novice.mp. [mp=title, abstract, heading word, drug trade name, original title, device manufacturer, drug manufacturer, device trade name, keyword heading word, floating subheading word, candidate term word]	12089

58	52 or 53 or 54 or 55 or 56 or 57	604441

59	51 and 58	2078

60	limit 59 to yr="2000 -Current"	1972

**CINAHL** **search strategy**

S1 (MH "Simulations")

S2 simulat* OR proxy for OR virtual realit* OR mixed realit* OR extended realit* OR XR OR mannekin* OR mannequin* OR avatar* OR role-play* OR visualisation* OR virtual* OR mock-up* OR imitat*

S3 train* OR educat* OR teach* OR learn*

S4 S2 AND S3

S5 S1 OR S4

S6 "nursing"

S7 nurs*

S8 S6 OR S7

S9 S5 AND S8

S10 "exam"

S11 exam*

S12 S10 OR S11

S13 "OSCE"

S14 competenc*

S15 S12 OR S13 OR S14

S16 S9 AND S15

S17 patient OR hospital OR clinic OR practice

S18 S16 AND S17

S19 trial* OR control* OR comparator* OR study OR test OR random* OR experiment* OR effect* OR comparison

S20 S18 AND S19

S21 student* OR pre-registration OR pre-licensure OR baccalaureate OR undergraduate OR novice

S22 S20 AND S21

S23 S20 AND S21

**PsychInfo search strategy**

S1 DE "Simulation"

S2 simulat* OR proxy for OR virtual realit* OR mixed realit* OR extended realit* OR XR OR mannekin* OR mannequin* OR avatar* OR role-play* OR visualisation* OR virtual* OR mock-up* OR imitat*

S3 train* OR educat* OR teach* OR learn*

S4 S2 AND S3

S5 S1 OR S4

S6 DE "Nursing"

S7 nurs*

S8 S6 OR S7

S9 S5 AND S8

S10 "exam"

S11 exam*

S12 S10 OR S11

S13 osce

S14 competenc*

S15 S12 OR S13 OR S14

S16 S9 AND S15

S17 patient OR hospital OR clinic OR practice

S18 S16 AND S17

S19 trial* OR control* OR comparator* OR study OR test OR random* OR experiment* OR effect* OR comparison

S20 S18 AND S19

S21 student* OR pre-registration OR pre-licensure OR baccalaureate OR undergraduate OR novice

S22 S20 AND S21

S23 S20 AND S21  Limited to Publication Year: 2000-2023

**Cochrane Central search strategy**

Date Run:	07/12/2022 14:38:36

#1	MeSH descriptor: [Simulation Training] explode all trees	1187

#2	simulat* OR proxy for OR virtual realit* OR mixed realit* OR extended realit* OR XR OR mannekin* OR mannequin* OR avatar* OR role-play* OR visualisation* OR virtual* OR mock-up* OR imitat*	58524

#3	train* OR educat* OR teach* OR learn*	255779

#4	#2 AND #3	18765

#5	#1 OR #4	18843

#6	nursing	45965

#7	nurs*	69037

#8	#6 OR #7	69037

#9	#5 AND #8	3102

#10	exam	6602

#11	exam*	244829

#12	#10 OR #11	244829

#13	OSCE	383

#14	competenc*	11819

#15	#12 OR #13 OR #14	253297

#16	#9 AND #15	1719

#17	patient OR hospital OR clinic OR practice	745575

#18	#16 AND #17	1521

#19	trial* OR control* OR comparator* OR study OR test OR random* OR experiment* OR effect* OR comparison	1973201

#20	#18 AND #19	1521

#21	student* OR pre-registration OR pre-licensure OR baccalaureate OR undergraduate OR novice	47049

#22	#20 AND #21 with Publication Year from 2000 to 2022, in Trials	299

**Figure S1. PRISMA flow diagram**

**Identification of studies via databases and registers**

Records removed *before screening*:

Duplicate records removed

(n = 4,466)

Records identified from

Databases (n = 9,159)

**Identification**

Records screened

(n = 4,693)

Records excluded

(n = 4,526)

Reports sought for retrieval

(n = 167)

Reports not retrieved

(n = 1)

**Screening**

Reports excluded: (n = 123)

Wrong participants (n =7)

Wrong intervention (n =3)

Wrong control (n =100)

Wrong outcomes (n=10)

Wrong study design (n=2)

Conference presentation (n=1)

Reports assessed for eligibility

(n = 166)

Studies included in review

(n = 42)

Reports of included studies

(n = 43)

**Included**

**Figure S2. Funnel plot of studies comparing simulation vs no simulation**

**References of included studies in the systematic review**

Alinier G, Hunt B, Gordon R, Harwood C. Effectiveness of intermediate-fidelity simulation training technology in undergraduate nursing education. *Journal of Advanced Nursing* 2006;54(3);359-369.

Ataee S, Aazami S, Direkvand-Moghadam A, Norozi S, Mozafari M. Nursing students' self-efficacy in cardiac critical care: a novel pre-internship educational package. *African Journal of Nursing and Midwifery*

2019;21(2);1-16

Bai P, Zang X, Liu R, Wang L, Dai C, Yang G. In-situ simulation for nursing students' professional competence development in postanesthesia care: A quasi-experimental study. *Nurse education in practice* 2023;70:103660.

Banjo-Ogunnowo SM, Chisholm LJ. Virtual versus traditional learning during COVID-19: quantitative comparison of outcomes for two articulating ADN cohorts. *Teaching and Learning in Nursing* 2022;17(3);272-276.

Cao l, Cao X. Application of staged simulated examination and mini-CEX in nursing practice teaching of obstetrics and gynaecology. Chinese Nursing Research 2015;29(8C):2964-7.

Centrella-Nigro AM, Blackwell B, Coughlin A, Voorhees KA. The effect of human patient simulators on knowledge and self-competence in graduating prelicensure nursing students. *Nursing Education Perspectives* 2016;37(6):337-339.

Craig SJ, Kastello JC, Cielowski BJ, Rovnyak V. Simulation strategies to increase nursing student clinical competence in safe medication administration practices: A quasi-experimental study. *Nurse Education Today* 2021;96;104605.

Curl ED, Smith S, Chisholm LA, McGee LA, Das K. Effectiveness of integrated simulation and clinical experiences compared to traditional clinical experiences for nursing students. *Nursing Education Perspectives* 2016;37(2) 72-77.

Dery SK, Kaufmann EE, Marzano D, Deininger M, Asem CK, Sienko KH. Design and evaluation of a subcutaneous contraceptive implant training simulator. *International Journal of Gynaecology and Obstetrics: The Official Organ of the International Federation Of Gynaecology and Obstetrics* 2019;147(1);36-42.

Guerrero JG, Hafiz AH, Eltohamy NA, Gomma N, Al Jarrah I. Repeated exposure to high-fidelity simulation and nursing interns' clinical performance: impact on practice readiness. *Clinical Simulation in Nursing* 2021;60;18-24.

Guerrero JG, Rosales NS, Castro GMT. Impact of high-fidelity simulation exposure of nursing students with their objective structured clinical examination: A quasi-experimental study. *Nursing Open* 2022;10:765–772.

Guerrero JG. A Longitudinal and Repeated Cross-sectional Study on the Effectiveness of the Transition of Clinical Teaching from Traditional to Simulation Era. *Clinical Simulation in Nursing* 2023;83:101444

Hall SW. High-fidelity simulation for senior maternity nursing students. *Nursing Education Perspectives* 2015;36(2);124-127.

Hansen J, Bratt M. Effect of sequence of simulated and clinical practicum learning experiences on clinical competency of nursing students. *Nurse Educator* 2017;42(5) 231-235.

Harris MA. Simulation-enhanced pediatric clinical orientation. *Journal of Nursing Education* 2011;50(8);461-465.

Hayden JK, Smiley RA, Alexander M, Kardong-Edgren S, Jeffries PR. The NCSBN National Simulation Study: A longitudinal, randomized, controlled study replacing clinical hours with simulation in prelicensure nursing education. *Journal of Nursing Regulation* 2014;5(2);S3-S40. AND Ingwerson J. National simulation study findings: Where do we go from here? Oregon State Board of Nursing Sentinel 2015;34(2):12-14

Hwang J, Park H. Comparison of the effects of standardized patient-based simulation education and clinical practice education for preoperative nursing care for high-risk pregnant women. *Medico-Legal Update* 2020;20(1);1900-1906.

Lee M, Ahn Y, Cho I, Sohn M. Effectiveness of simulation integrated with problem based learning on clinical competency and self-efficacy in nursing students. Child Health Nursing Research 2014;20(2):123-131

Lee J, Son HK. Effects of electronic fetal monitoring simulation with problem-based learning on nursing students' performance confidence, clinical judgment, and knowledge. *Japan journal of nursing science: JJNS* 2023;20(4):e12539.

Luctkar-Flude M, Wilson-Keates B, Larocque M. Evaluating high-fidelity human simulators and standardized patients in an undergraduate nursing health assessment course. *Nurse Education Today* 2012;32(4);448-452.

Mancini ME, LeFlore JL, Cipher DJ. Simulation and clinical competency in undergraduate nursing programs: a multisite prospective study. *Journal of Nursing Education* 2019;58(10);561-568.

Meyer MN, Connors H, Hou Q, Gajewski B. The effect of simulation on clinical performance: A junior nursing student clinical comparison study. *Simulation in Healthcare* 2011;6(5);269-277.

Olaussen C, Steindal SA, Jelsness‑Jorgensen L-P, Aase I, Stenseth HV, Tvedt CR. Integrating simulation training during clinical practice in nursing homes: an experimental study of nursing students' knowledge acquisition, self-efficacy and learning needs. *BMC Nursing* 2022;21(47);1-16

O’Lynn C, Krautscheid L. Evaluating the effects of intimate touch instruction: Facilitation professional and respectful touch by male nursing students. *Journal of Nursing Education* 2014;53(3):126-135

Radnakrishnan K, Roche JP, Cunningham H. Measuring clinical practice parameters with human patient simulation: A pilot study. I*nternational Journal of Nursing Education Scholarship* 2007;4(1):1-11

Raman S, Labrague LJ, Arulappan J, Natarajan J, Amirtharaj A, Jacob D. . Traditional clinical training combined with high-fidelity simulation-based activities improves clinical competency and knowledge among nursing students on a maternity nursing course. *Nursing Forum* 2019;54(3);434-440.

Reaves RP, McEwing EM, Matsuda Y, Everett-Thomas R. Using simulation to evaluate exam scores in a public health nursing course. *Public Health Nursing* 2020;37:596–601

Reid CA, Ralph JL., El-Masri M, Ziefle K. High-fidelity simulation and clinical judgment of nursing students in a maternal-newborn course. *Western Journal of Nursing Research* 2020;42(10);829-837.

Roberts S, Warren T, Moore LC. COVID-19 pandemic: effects of replacing clinical hours with virtual simulation in BSN prelicensure nursing education. *Nursing Education Perspectives* 2022;43(5);306-308.

Schlairet MC, Pollock JW. Equivalence testing of traditional and simulated clinical experiences: Undergraduate nursing students' knowledge acquisition. *Journal of Nursing Education* 2010;49(1);43-47.

Sears K, Goldsworthy S, Goodman WM. The relationship between simulation in nursing education and medication safety. *Journal of Nursing Education* 2010;49(1);52-55.

Seo YH, Eom MR. The effect of simulation nursing education using the outcome-present state-test model on clinical reasoning, the problem-solving process, self-efficacy, and clinical competency in Korean nursing students. *Healthcare (Basel, Switzerland)* 2021;9(243);1-13

Soccio DA. Effectiveness of mental health simulation in replacing traditional clinical hours in baccalaureate nursing education. *Journal of Psychosocial Nursing and Mental Health Services* 2017;55(11);36-43.

Son HK. Effects of s-pbl in maternity nursing clinical practicum on learning attitude, metacognition, and critical thinking in nursing students: A quasi-experimental design. *International Journal of Environmental Research and Public Health* 2020;17(21);1-12.

Tawalbeh LI. Effect of simulation modules on Jordanian nursing student knowledge and confidence in performing critical care skills: A randomized controlled trial. *International Journal of Africa Nursing Sciences* 2020;13;100242.

Terzioglu F, Yücel C, Koc G, Sinsek S, Yasar BN, Sahan FU, Akin R. et al. A new strategy in nursing education: From hybrid simulation to clinical practice. *Nurse Education Today* 2016;39;104-108.

Thomas CM, Barker N. Impact of simulation on undergraduate student outcomes. *Nurse Educator* 2022;47(6) e127-e131.

White P, Champion JD. Transitioning undergraduate maternal-newborn and pediatric hospital clinical experience to immersive simulation-based education. *Clinical Simulation in Nursing* 2021;61;10-13.

Witt MA, McGaughan K, Smaldone A. Standardized patient simulation experiences improves mental health assessment and communication. *Clinical Simulation in Nursing* 2018;23;16-20.

Woda A, Schnable T, Alt-Gehrman P, Bratt MM, Garnier-Villarreal M. Innovation in clinical course delivery and impact on students' clinical decision-making and competence. *Nursing Education Perspectives* 2019;40(4);241-243.

Yu M, Kang KJ. Effectiveness of a role-play simulation program involving the sbar technique: A quasi-experimental study. *Nurse Education Today* 2017;53;41-47.

Yu M, Yang M, Ku B, Mann JS. Effects of virtual reality simulation program regarding high-risk neonatal infection control on nursing students. *Asian Nursing Research* 2021;15(3);189-196.

**Table S1. List of excluded studies and reasons for exclusion**

|  | Reference | Reason |
| --- | --- | --- |
| 1. | Aebersold M, Voepel-Lewis T, Cherara L, Weber M, Khouri C, Levine R, Tait AR. Interactive anatomy-augmented virtual simulation training. Clinical Simulation in Nursing 2018;15:34-41 | No patient-based education control group |
| 2. | Aggar C, Bloomfield JG, Frotiold A, Thomas TH, Koo F. A time management intervention using simulation to improve nursing students’ preparedness for medication administration in the clinical setting: A quasi-experimental study. Collegian 2018;25:105–111 | No patient-based education control group |
| 3. | Ahn MK, Lee CM. Development and Effects of Head-Mounted Display-Based Home-Visits Virtual Reality Simulation Program for Nursing Students. Journal of the Korean Academy of Nursing 2021;51(4):465 | No patient-based education control group |
| 4. | Alinier G, Hunt WB, Gordon R. Determining the value of simulation in nurse education: study design and initial results. Nurse Education in Practice 2004;4:200–207 | Can’t tell what education the control group received |
| 5. | Alkalaf AA, Wazqar DY. The effect of high-fidelity simulation technology on the competency of nursing students in managing chemotherapy extravasation in patients with cancer. Journal of Professional Nursing 2022;42:1–7 | No patient-based education control group |
| 6. | Anon. The use of immersive and virtual reality technologies to enable nursing students to experience scenario-based, basic life support training—exploring the impact on confidence and skills. CIN: Computers, Informatics, Nursing. 2020;38(6):320-1 | Test of understanding about simulation teaching and learning |
| 7. | Aqel AA, Ahmad MM. High-fidelity simulation effects on CPR knowledge, skills, acquisition, and retention in nursing students. Worldviews on Evidence-Based Nursing, 2014;11:6:394–400. | Both groups received simulation training |
| 8. | Arabpur A, Farsi Z, Butler S, Habibi H. Comparative effectiveness of demonstration using hybrid simulation versus task-trainer for training nursing students in using pulse-oximeter and suction: A randomized control trial. Nurse Education Today 2022;110:105204 | Control group received no training (simulation group had extra training) |
| 9. | Aronson B, Glynn B, Squires T. Competency assessment in simulated response to rescue events. Clinical Simulation in Nursing 2012;8:e289-e295 | No patient-based education control group |
| 10. | Arslan FT, Turkmen AS, Celen R, Ozkan S, Altiparmak D, Sahin A. Comparing traditional and simulation-based experiences in pediatrics with undergraduate nursing students in Turkey Clinical Simulation in Nursing 2018;16:62-69 | No competency measures |
| 11. | Avraham R, Cohen T, Artzi-Medvedik R, Hurvitz N, Cohen O. Effectiveness of a virtual program for OSCE preparation during COVID-19: a descriptive and repeated cross-sectional study among nursing students. BMC Nursing. 2023;22(1). | Intervention OSCE preparation |
| 12. | Azizi M, Ramezani G, Karimi E, Hayat AA, Faghihi SA, Keshavarzi MH. A comparison of the effects of teaching through simulation and the traditional method on nursing students’ self‑efficacy skills and clinical performance: a quasi‑experimental study. BMC Nursing 2022;21:283 https://doi.org/10.1186/s12912-022-01065-z | No patient-based education control group |
| 13. | Baginski MN. Use of low-fidelity simulation within a medication calculation nursing course to enhance students’ understanding of accuracy. Nurse Educator 2017;42(3):110-111 | No patient-based education control group |
| 14. | Baillie L, Curzio J. Students’ and facilitators’ perceptions of simulation in practice learning. Nurse Education in Practice 2009;9:297–306 | Subjective outcomes only, no objective outcome measures |
| 15. | Ballard G, Piper S, Stokes P. Effect of simulated learning on blood pressure measurement skills. Nursing Standard. 2012;27(8):43-47. | Control group received no training (simulation group had extra training) |
| 16. | Bearnson CS, Wiker KM. Human patient simulators: a new face in baccalaureate nursing education at Brigham Young University. Journal of Nursing Education 2005;44(9):421-5 | No patient-based education control group |
| 17. | Beddingfield S, Davis BW, Gilmore M, Jenkins L. The effect of high fidelity simulation on examination performance. Teaching and Learning in Nursing 2011;6:46-9 | No numerical results per group given |
| 18. | Blum CA, Borglund S, Parcells D. High-fidelity nursing simulation: impact on student self-confidence and clinical competence. International Journal of Nursing Education Scholarship 2010  DOI: 10.2202/1548-923X.2035 | No patient-based education control group |
| 19. | Bornais JA, Raiger JE, Krahn RE, El-Masri MM.  Evaluating undergraduate nursing students' learning using standardized patients. Journal of Professional Nursing 2012;28:291–296 | No patient-based education control group |
| 20. | Bryant R, Miller CL, Henderson D. Virtual clinical simulations in an online advanced health appraisal course. Clinical Simulation in Nursing 2015;11:437-444 | No patient-based education control group |
| 21. | Chang Y-L, Hsieh M-J, Feng T-H, Shang S-T, Tsai Y-F. Effectiveness of multiple scenario simulations of acute and critical care for undergraduate nursing students: A quasi-experimental design. Nurse Education Today 2022;118:105526 | No patient-based education control group |
| 22. | Chen J, Yang J, Hu F, Yu S-H, Yang B-X, Liu Q et al. Standardised simulation-based emergency and intensive care nursing curriculum to improve nursing students’ performance during simulated resuscitation: A quasi-experimental study. Intensive & Critical Care Nursing 2018;46:51–56 | No patient-based education control group |
| 23. | Chen H-M, Liu H-S, Chao S-Y. The effects of web-based situational learning on nursing students' transfer of learning in clinical practice. Nurse Education Today 2021;105:105052 | No patient-based education control group |
| 24. | Chen T-J, Traynor V, Ho M-H, Chang H-CR, Rolls K, Pratt H, et al. Effects of simulation-based education module on delirium care in undergraduate nursing students: A quasi-experimental study. Nurse education in practice. 2023;74:103852. | No patient-based education control group |
| 25. | Chen Y, Li X, Zhao J, Li S, Dai Y, Zhou Z. Effects of the mini-clinical evaluation exercise teaching mode based on nurse-faculty cooperation in Fundamentals of Nursing course: A quasi-experimental study. International journal of nursing sciences. 2023;10(3):359-66. | No patient-based education control group |
| 26. | Chircop A, Cobbett S. Gett’n on the bus: evaluation of Sentinel City®3.0 virtual simulation in community/ population health clinical placement. International Journal of Nursing Education and Scholarship 2020;20190109 doi.org/10.1515/ijnes-2019-0109 | Subjective outcomes only, no objective outcome measures |
| 27. | Choi H, Lee U, Jeon YS, Kim C. Efficacy of the computer simulation-based, interactive communication education program for nursing students. Nurse Education Today 2020;91:104467 | No patient-based education control group |
| 28. | Choi H, Jeon Y, Lee U, Ahn J, Kim H. Technology-based interactive communication simulation for Korean nurses: A randomized controlled repeated-measures design. Nurse education today. 2023;128:105879. | Participant group qualified nurses |
| 29. | Coggins AR, Nottingham C, Byth K, Ho KR, Aulia FA, Murphy M et al. Randomised controlled trial of simulation-based education for mechanical cardiopulmonary resuscitation training. Emergency Medicine Journal 2019;36:266–272 | No patient-based education control group |
| 30. | Cook NF, McAloon TM, O’Neill P, Beggs R. Impact of a web based interactive simulation game (PULSE) on nursing students' experience and performance in life support training — A pilot study. Nurse Education Today 2012;32:714–720 | No patient-based education control group |
| 31. | Cooper EE, Prion SK, Pauly-O’Neill SJ. Comparison of student experience with critical events during simulation and acute care hospital rotations. Nurse Educator 2015;40(1):31-35 | No patient-based education control group |
| 32. | Diaz DA, Eckhoff DO, Nunes M, Anderson M, Keiffer M, Salazar I et al. Discovery of methods to enhance the care of the LGBTQ+ community. The Journal for Nurse Practitioners 2021;17:1085e1090 | No comparative results of simulation vs patient-based training |
| 33. | Edeer AD, Vural F, Damar HT, Yasak K, Damar M. The effect of web-based preoperative and postoperative patient care education on nursing students. A randomized controlled study. CIN: Computers, Informatics, Nursing 2019;37(10):541-7 DOI: 10.1097/CIN.0000000000000552 | No patient-based education control group |
| 34. | Eghbalibabadi M, Ashouri E. Comparison of the effects of two teaching methods on the nursing students’ performance in measurement of blood pressure. Iranian Journal of Nursing and Midwifery Research 2014;19(4):381-4 | No patient-based education control group |
| 35. | Eom M-R, Kim H-S, Kim E-K, Seong K. Effects of teaching method using standardized patients on nursing competence in subcutaneous injection, self-directed learning readiness and problem solving ability. Journal of the Korean Academy of Nursing 2010;40(2):151-160 | No patient-based education control group |
| 36. | Erenel AS, Sozbir SY, Aksoy MU, GÜRCÜOĞLU AE, Aksu SP, Toprak FU et al. Effect of scenario-based simulation training on the obstetrics and gynecology nursing clinical practicum. The Journal of Nursing Research 2021;29(2):1-9 | Subjective outcomes only. No measures of clinical ability |
| 37. | Fawaz MA, Hamdan-Mansour AM. Impact of high-fidelity simulation on the development of clinical judgment and motivation among Lebanese nursing students. Nurse Education Today 2016;46:36–42 | No patient-based education control group |
| 38. | Fugslang S, Bloch CW, Selberg H. Simulation training and professional self-confidence: A large-scale study of third year nursing students. Nurse Education Today 2022;108:105175 | Both groups received simulation training |
| 39. | Gates MG, Parr MB, Hughen JE. Enhancing nursing knowledge using high-fidelity simulation. Journal of Nursing Education 2012;51(1):9-15 | Both groups received simulation training |
| 40. | Goldsworthy S, Patterson JD, Dobbs M, Afzal A, Seboer S. How does simulation impact building competency and confidence in recognition and response to the adult and paediatric deteriorating patient among undergraduate nursing students? Clinical Simulation in Nursing 2019;28:25-32 | Training control group received unclear |
| 41. | Goldsworthy S, Muir N, Baron S, Button D, Goodhand K, Hunter S et al. The impact of virtual simulation on the recognition and response to the rapidly deteriorating patient among undergraduate nursing students. Nurse Education Today 2022;110:105264 | Training control group received unclear |
| 42. | Habibli T, Ghezeljeh TN, Haghani S. The effect of simulation-based education on nursing students’ knowledge and performance of adult basic cardiopulmonary resuscitation: A randomized clinical trial. Nursing Practice Today 2020;7(2):87-96 | No patient-based education control group |
| 43. | Haddeland K, Slettebo A, Svensson E, Tosterud RB, Wangensteen S, Fossum M. The effects of using high-fidelity simulation in undergraduate nursing education: A multicenter randomized controlled trial with a process evaluation. International Journal of Educational Research 2021;109:101813 | No patient-based education control group |
| 44. | Haerling K, Kmail Z, Buckingham A. Contributing to Evidence-Based Regulatory Decisions: A Comparison of Traditional Clinical Experience, Mannequin-Based Simulation, and Screen-Based Virtual Simulation. Journal of Nursing Regulation. 2023;13(4):33-43. | Both groups received simulation |
| 45. | Hester L, Reed B, Bohannan W, Box M, Wells M, O’Neal B. Using an educational mobile application to teach students to take vital signs. Nurse Education Today 2021;107:105154 | No patient-based education control group |
| 46. | Howard VM, Ross C, Mitchell AM, Nelson GM. Human Patient Simulators and Interactive Case Studies: A Comparative Analysis of Learning  Outcomes and Student Perceptions. CIN: Computers, Informatics, Nursing 2010;28(1)42–48 | No patient-based education control group |
| 47. | Hsu L-L, Chang W-H, Hsieh S-I. The effects of scenario-based simulation course training on nurses' communication competence and self-efficacy: A randomized controlled trial. Journal of Professional Nursing 2015;31:37–49 | No patient-based education control group |
| 48. | Hur HK, Roh YS. Effects of a Simulation based Clinical Reasoning Practice Program on Clinical Competence in Nursing Students. Korean Journal of Adult Nursing 2013;25(5):574-584 | No patient-based education control group |
| 49. | Ignacio J, Dolmans D, Scherpbier A, Rethans J-J, Chan S, Liaw SY. Comparison of standardized patients with high-fidelity simulators for managing stress and improving performance in clinical deterioration: A mixed methods study. Nurse Education Today 2015;35:1161–1168 | Standard patient vs simulation |
| 50. | Ironside PM, Jeffries PR. Using multiple-patient simulation experiences to foster clinical judgment. Journal of Nursing Regulation 2010;1(2):38-41 | No patient-based education control group |
| 51. | Jansson MM, Ala-Kokko TI, Ohtonen PP, Merilainen MH, Syrjala HP, Kyngas HA. Human patient simulation education in the nursing management of patients requiring mechanical ventilation: A randomized, controlled trial. American Journal of Infection Control 2014;42:271-6 | Training control group received unclear |
| 52. | Jarvill M, Jenkins S, Akman O, Astroth KS, Pohl C, Jacobs PJ. Effect of simulation on nursing students’ medication administration competence. Clinical Simulation in Nursing 2018;14:3-7 | No patient-based education control group |
| 53. | Joo, GE, Sohng K-Y, Kim H-J. Effects of a Standardized patient simulation program for nursing students on nursing competence, communication skill, self-efficacy and critical thinking ability for blood transfusion. Journal of the Korean Academy of Fundamentals in Nursing 2015;22(1):49-58 | No patient-based education control group |
| 54. | Kang K-A, Kim S, Kim S-J, Oh J, Lee M. Comparison of knowledge, confidence in skill performance (CSP) and satisfaction in problem-based learning (PBL) and simulation with PBL educational modalities in caring for children with bronchiolitis. Nurse Education Today 2015;35:315–321 | No patient-based education control group |
| 55. | Karkada S, Radhakrishnan J, Natarajan J, Matua GA, Kaddoura M, Knowledge and competency of novice nursing students in nasogastric tube feeding: Is simulation better than case scenario? Oman Medical Journal 2019;34(6):528-533 | No patient-based education control group |
| 56. | Khraisat AM, Hapadin H, Ahmad NS, Yusoff MD, Nurumal MS, Pardi W. How to teach intramuscular injection through virtual learning environment in covid-19 pandemic time for nursing students. Indian Journal of Forensic Medicine & Toxicology 2020;14(4):697-704 | No patient-based education control group |
| 57. | Kim S, Shin G. Effects of nursing process-based simulation for maternal child emergency nursing care on knowledge, attitude, and skills in clinical nurses. Nurse Education Today 2016;37:59–65 | Participants are qualified nurses |
| 58. | Kim J, Heo N. Effect of a Simulated Education-based Hypoglycemia Scenario Using a High-fidelity Simulator on Acquisition and Retention of Diabetes Knowledge and Academic Self-efficacy in Nursing Students. Journal of the Korean Academy of Social Nurse Education 2017;23(3):319-329 | No patient-based education control group |
| 59. | Kim M-G, Kim H-W. The effects of classes using virtual reality simulations of the hospital environment on knowledge of the hospital environment, academic self-efficacy, learning flow, educational satisfaction and academic achievement in nursing students. Journal of the Korean Academy of Fundamental Nursing 2021;28(4):520-529 | No patient-based education control group |
| 60. | Kim Y-E, Kang H-Y. Effects of a Nursing Simulation Learning Module on Clinical Reasoning Competence, Clinical Competence, Performance Confidence, and Anxiety in COVID-19 Patient-Care for Nursing Students. Journal of Korean Academy of Nursing. 2023;53(1):87-100. | No patient-based education control group |
| 61. | Ko S, Choi E-H. Effect of team debriefing in simulation-based cardiac arrest emergency nursing education. Korean Journal of Adult Nursing 2017;29(6):667-676 | No patient-based education control group |
| 62. | Koo HY, Lee BR. Development and evaluation of a pediatric nursing competency-building program for nursing students in South Korea: a quasi-experimental study. Child Health Nursing Research, 2022;28(3):167-175 | Training control group received unclear |
| 63. | Lee M-N, Kang K-A, Park S-J, Kim S-J. Effects of pre-education combined with a simulation for caring for children with croup on senior nursing students. Nursing and Health Sciences 2017;19:264–272 | No patient-based education control group |
| 64. | Lee B-O, Liang H-F, Chu T-P, Hung C-C. Effects of simulation-based learning on nursing student competences and clinical performance. Nurse Education in Practice 2019;41:102646 | Cannot tell whether the control group received patient-based teaching |
| 65. | Lee H, Han J-W. Development and evaluation of a virtual reality mechanical ventilation education program for nursing students. BMC Medical Education 2022;22:775 https://doi.org/10.1186/s12909-022-03834-5 | No patient-based education control group |
| 66. | Lee JS. Implementation and evaluation of a virtual reality simulation: Intravenous injection training system. International Journal of Environmental Research and Public Health 2022;19:5439. https://doi.org/10.3390/ijerph19095439 | No patient-based education control group |
| 67. | Lee E, Baek G. Development and Effects of a Virtual Reality Simulation Nursing Education Program Combined With Clinical Practice Based on an Information Processing Model. Computers, informatics, nursing : CIN. 2023;41(12):1016-25. | Intervention |
| 68. | Lee E, Baek G, Hwang Y. Effectiveness of the Patient's Severity Classification Competency Promotion Virtual Reality Program of Nursing Students during the COVID-19 Pandemic Period. Healthcare (Basel, Switzerland). 2023;11(8) | No patient-based education control group |
| 69. | Lee Min H, Noh E-Y. Effectiveness of Simulation-Based Education for Caring Patients with COVID-19. Journal of Korean Academy of Nursing. 2023;53(4):397-411. | No patient-based education control group |
| 70. | Legge TR, Holthaus A, Hallmark B, Alexander K. Preparing for the Next Generation NCLEX Using a Virtual Simulation. The Journal of nursing education. 2023:1-5 | No patient-based education control group |
| 71. | Li N, Chen HL, Li MJ, Huang YQ, Li HS, Wang LH. Prospective study on application of mind mapping combined with scenario simulation training on the ability training of junior nurses in hospital transfer of patients with critical burns and trauma. Chinese journal of burns. 2023;39(5):465-71. | Participant group qualified nurses |
| 72. | Liaw SY, Rethans J_J, Scherpbier A, Klainin-Yobas P. Rescuing a patient in deteriorating situations (RAPIDS): a simulation-based educational program on recognizing, responding and reporting of physiological signs of deterioration. Resuscitation 2011;82:1224– 1230 | No information on education that control group received |
| 73. | Liaw SY, Scherpbier A, Rethans J_J, Klainin-Yobas P. Assessment for simulation learning outcomes: A comparison of knowledge and self-reported confidence with observed clinical performance. Nurse Education Today 2012;32:e35–e39 | Control group received no training (simulation group had extra training) |
| 74. | Mather C, McCarthy R. Exploring the effects of a high-fidelity environment on nursing students’ confidence and performance of CPR. Nursing Standard. 2021 doi: 10.7748/ns.2021.e11564 | Both groups received simulation training |
| 75. | McIntosh KS, Gregor JC, Khanna NV. Computer-based virtual reality colonoscopy simulation improves patient-based colonoscopy performance. Canadian Journal of Gastroenterology and Hepatology 2014;28(4):203-206. | Participants were not nurses |
| 76. | Meny A, Hayat A, Eldigire M, Kaleem M, Alharbi N, Albaz N, et al. Comparing the Effectiveness of Role-Play Simulation versus Real Patient Transferal Skills Training in Occupational Therapy Students in Saudi-Arabia- A Quasi-Experimental Study. ADVANCES IN MEDICAL EDUCATION AND PRACTICE. 2023;14:685-91 | Participant group occupational therapists |
| 77. | Miranda RP, Chaves ED, Lima RS, Braga CG, Simoes IA, Fava SM et al. The effectiveness of a simulated scenario to teach nursing students how to perform a bed bath: A randomized clinical trial. Nurse Education Today 2017;57:17–23 | Standard patient vs simulation |
| 78. | Mohammed Salem A, Ahmed Mohamed NM, Masadeh A. The effectiveness of computer-based simulation on nursing students' electrocardiogram interpretation. Teaching & Learning in Nursing. 2023;18(3):e9-e12. | No patient-based education control group |
| 79. | Murphy LJ, Janisse L. Optimizing transition to practice through orientation: A quality improvement initiative Clinical Simulation in Nursing 2017;13:583-590 | Control group did not receive patient-based education |
| 80. | Onarici M, Karadag M. The effect of simulation method on nursing students’ burn patient care planning: A randomized controlled trial. Journal of Burn Care & Research 2021;42(5):1011-6 doi:10.1093/jbcr/irab018 | Control group received no training (simulation group had extra training) |
| 81. | Otero-Agra M, Hermo-Gonzalo MT, Santos-Folgar M, Fernandez-Mendez F, Barcala-Furelos R. Assessing ventilation skills by nursing students in paediatric and adult basic life support: A crossover randomized simulation study using bag-valve-mask (BMV) vs mouth-to-mouth ventilation (MMV). Signa Vitae 2020;16(2):44-51 | Both groups received simulation training |
| 82. | Palliyalil S, Bashir RA, Zein H, Murthy P, Flores-Sarnat L, Sarnat HB, et al. The Utility of Simulation-Based Training in Teaching Frontline Providers Modified Sarnat Encephalopathy Examination: A Randomized Controlled Pilot Trial. Pediatric neurology. 2023;139:70-5 | Participant group qualified nurses |
| 83. | Park J, Kim K-J. Effects of patient deterioration simulation using inattentional blindness for final year nursing students: A randomized controlled trial. Nurse Education Today 2021;106:105080 | Both groups received simulation training |
| 84. | Park S-A, Kim HY. Development and effects of a labor nursing education program using a high-fidelity simulator for nursing students. Korean Journal of Women Health Nursing 2020;26(3):240-249 | No patient-based education control group |
| 85. | Paul C, Bhokare N, Pathak S. Investigate the effect of role play on the skill among 2nd basic B.Sc. students if SRMMCON regarding selected community procedure. Indian Journal of Forensic Medicine & Toxicology, Oc tober-December 2020, Vol. 14, No. 4. 6457-60 | No simulation used |
| 86. | Rashwan ZI. Motivation inspiring confidence: effect of scenario-based learning on self-confidence among prelicensure nursing students. Teaching & Learning in Nursing. 2023;18(3):e1-e8. | Subjective outcomes only, no objective outcome measures |
| 87. | Ross JG. The Effect of Simulation Training on Baccalaureate Nursing Students’ Competency in Performing Intramuscular Injection. Nursing Education Perspectives 2015;36(1):48-9 doi: 10.5480/13-1075.1 | No patient-based education control group |
| 88. | Rutty J, Biggs M, Dowsett D, Kitchener A, Coltman N, Rutty G. Post mortem computed tomography: An innovative tool for teaching anatomy within pre-registration nursing curricula. Nurse Education Today 2019;76:154–164 | No patient-based education control group |
| 89. | Ryu J, Yu M. Virtual Reality Simulation for Advanced Infection Control Education in Neonatal Intensive Care Units: Focusing on the Prevention of Central Line-Associated Bloodstream Infections and Ventilator-Associated Infections. Healthcare (Basel, Switzerland). 2023;11(16). | Participant group qualified nurses |
| 90. | Salameh B, Ayed A, Kassabry M, Lasater K. Effects of a complex case study and high-fidelity simulation on mechanical ventilation on knowledge and clinical judgment of undergraduate nursing students. Nurse Educator 2021;46(4):E64-E69. doi: 10.1097/NNE.0000000000000938 | No patient-based education control group |
| 91. | Sarmasoglu S, Dinc L, Elcin M. Using Standardized Patients in Nursing Education Effects on Students’ Psychomotor Skill Development. Nurse Educator 2016;41(2):E1-E5 | Standard patient vs simulation |
| 92. | Sarvan S, Efe E. The effect of neonatal resuscitation training based on a serious game simulation method on nursing students' knowledge, skills, satisfaction and self-confidence levels: A randomized controlled trial. Nurse Education Today 2022;111:105298 | No patient-based education control group |
| 93. | Secomb J, McKenna L, Smith C. The effectiveness of simulation activities on the cognitive abilities of undergraduate third-year nursing students: a randomised control trial. Journal of Clinical Nursing 2012;21:3475–3484, doi: 10.1111/j.1365-2702.2012.04257.x | Both groups received simulation training |
| 94. | Sezgunsay E, Basak T. Is moulage effective in improving clinical skills of nursing students for the assessment of pressure injury? Nurse Education Today 2020;94:104572 | Both groups received simulation training |
| 95. | Shinnick MA, Woo MA. Does nursing student self-efficacy correlate with knowledge when using human patient simulation? Clinical Simulation in Nursing 2014;10:e71-e79 | No patient-based education control group |
| 96. | Silva JL, Kumakura AR, Zanchetta FC, Lima MH. Clinical simulation for teaching of wound evaluation and treatment. Clinical Simulation in Nursing 2020;38:5-13 | No patient-based education control group |
| 97. | Smith AS, Lollar J, Mendenhall J, Brown H, Johnson P, Roberts S. Use of multiple pedagogies to promote confidence in triage decision making: A pilot study. Journal of Emergency Nursing 2013;39(6):660-666. | No patient-based education control group |
| 98. | Smith SJ, Barry DG. An innovative approach to preparing nursing students for care of the elderly in the home. Geriatric Nursing 2013;34:30e34 | No information on education that control group received |
| 99. | Smith SJ, Farra S, Ulrich DL, Hodgson E, Nicely S, Matcham W. Learning and retention using virtual reality in a decontamination simulation. Nursing Education Perspectives 2016;37(4):210-14 | No patient-based education control group |
| 100. | Smith SJ, Farra S, Ulrich DL, Hodgson E, Nicely S, Mickle A. Effectiveness of two varying levels of virtual reality simulation. Nursing Education Perspectives 2018;39(6):E10-E15 | No patient-based education control group |
| 101. | Son HK, Kim DH. Effect of SEGUE-based communication education on nursing simulation practice: a quasi-experimental design. Contemporary Nurse, 2019;55(4–5:330–340, doi.org/10.1080/10376178.2019.1641421 | Both groups received simulation training |
| 102. | Sparacino, L. Della Vecchia, E. Using high- fidelity simulation to close the teaching and learning gap. Online Journal of Nursing Informatics (OJNI), 2013;17(1):2392 | No patient-based education control group |
| 103. | Speeney N, Kameg KM, Cline T, Szpak JL, Bagwell B. Impact of a standardized patient simulation on undergraduate nursing student knowledge and perceived competency of the care of a patient diagnosed with schizophrenia. Archives of Psychiatric Nursing 2018;32:845–849 | No patient-based education control group |
| 104. | Svellingen AH, Forstronen A, Assmus J, Roykenes K, Brattebo G. Simulation-based education and the effect of multiple simulation sessions - A randomised controlled study. Nurse Education Today 2021;106:105059 | Both groups received simulation training |
| 105. | Svellingen AH, Forstronen A, Assmus J, Roykenes K, Brattebo G. Examining predictive factors of nursing students’ self-confidence in multiple simulation sessions: A randomized controlled study. Nurse Education in Practice 2021;57:103231 | Both groups received simulation training |
| 106. | Takhdat K, Eddabbah M, Hamzaoui H, Lamtali S, Adib AR. High-fidelity simulation effects on cardiopulmonary resuscitation self-efficacy and knowledge retention in undergraduate nursing students: A Two-Group, Experimental, Longitudinal Pilot Study. Nursing Education Perspectives 2022;43(6):E118-20 | No patient-based education control group |
| 107. | Tamaki T, Inumaru A, Yokoi Y, Fuji M, Tomita M, Inoue Y et al. The effectiveness of end-of-life care simulation in undergraduate nursing education: A randomized controlled trial. Nurse Education Today 2019;76:1–7 | No patient-based education control group |
| 108. | Tan AJ, Lee CC, Lin PY, Cooper S, Lau LS, Chua WL et al. Designing and evaluating the effectiveness of a serious game for safe administration of blood transfusion: A randomized controlled trial. Nurse Education Today 2017;55:38–44 | Control group received no training (simulation group had extra training) |
| 109. | Tarhan M, Yildirim A. Effect of Repeated Multipatient Simulations on Professional Readiness Among Senior Nursing Students. Nurse educator. 2023;48(4):197‐203. | No patient-based education control group |
| 110. | Thomas C, Mackey E. Influence of a clinical simulation elective on baccalaureate nursing student clinical confidence. Journal of Nursing Education 2012;51(4):236-9 | Subjective outcomes only, no objective outcome measures. No measures of clinical ability |
| 111. | Tuzer H, Dinc L, Elcin M. The effects of using high-fidelity simulators and standardized patients on the thorax, lung, and cardiac examination skills of undergraduate nursing students. Nurse Education Today 2016;45:120–125 | Standard patient vs simulation |
| 112. | Tuzer H, Yilmazer T. Standardized patient education in nursing students’ effect of postoperative care management. Turkiye Klinikleri Journal of Nursing Science. 2020;12(3):366-70 | Control group received no training (simulation group had extra training) |
| 113. | Unal E, Ozdemir A. The effect of hybrid simulated burn care training on nursing students' knowledge, skills, and empathy: A randomised controlled trial. Nurse education today. 2023;126:105828. | No patient-based education control group |
| 114. | Uzen Cura S, Kocatepe V, Yildirum D, Küçükakgün H, Atay S, Unver V. Examining knowledge, skill, stress, satisfaction, and self-confidence levels of nursing students in three different simulation modalities. Asian Nursing Research 2020;14:158e164 | No patient-based education control group |
| 115. | Veltri LM, Rowe JM, Bell KJ, Arwood EL, Kindler LL. The maternal-newborn assessment study: can simulation replicate the clinical learning experience in undergraduate nursing education? JOGNN, 43, S81-S85; 2014. DOI: 10.1111/1552-6909.12438 | Conference presentation |
| 116. | Vural Dogru B, Aydin LZ. The effects of training with simulation on knowledge, skill and anxiety levels  of the nursing students in terms of cardiac auscultation: A randomized controlled study. Nurse Education Today 2020;84:104216 | Both groups received simulation training |
| 117. | Wang MY, Chen XH, Yang YW, Wang HY, Yan Y, Huang XY, et al. Effect evaluation of case-based learning with situated cognition theory on competence training for student nurses in pediatric surgery. HELIYON. 2023;9(2). | No patient-based education control group |
| 118. | Woda A, Hansen J, Paquette M, Topp R. The impact of simulation sequencing on perceived clinical decision making. Nurse Education in Practice 2017;26:33e38 | Randomised crossover trial of simulation vs hospital training but no midpoint assessment |
| 119. | Wong FMF, Chan AML, Lee NPM, Luk KKH. Can High-Fidelity Patient Simulation Be Used for Skill Development in Junior Undergraduate Students: A Quasi-Experimental Study. Healthcare (Basel, Switzerland). 2023;11(15). | Subjective outcomes only, no objective outcome measures |
| 120. | Xi S, Li L, Wei H, Gu Y. Application of evidence-based scene simulation combined with problem-based learning in clinical teaching of critical care for nursing students. Chinese Journal of Integrative Nursing 2022;8(3):19-22 | No patient-based education control group |
| 121. | Yang F, Wang Y, Yang C, Zhou MH, Shu J, Hu H. Improving clinical judgment by simulation: A randomized trial and validation of the Lasater clinical judgment rubric in Chinese. BMC Medical Education 2019;19:20 https://doi.org/10.1186/s12909-019-1454-9 | No patient-based education control group |
| 122. | Yilmaz DU, Sari D. Examining the effect of simulation-based learning on intravenous therapy administration’ knowledge, performance, and clinical assessment skills of first-year nursing students. Nurse Education Today 2021;102:104924 | Both groups received simulation training |
| 123. | Yong Y, Ying Z. Application of emergency care simulator in the teaching of clinical skills for nursing undergraduate students. Nursing of Integrated Traditional Chinese and Western Medicine 2020;6(5):193-5 | No patient-based education control group |

**Quality assessment checklist results**

**Table S2. RCT and randomised crossover trial results**

|  | **Q1** | **Q2** | **Q3** | **Q4** | **Q5** | **Q6** | **Q9** | **Q10** | **Q11** | **Comment** |
| --- | --- | --- | --- | --- | --- | --- | --- | --- | --- | --- |
| Alinier 2006 | Y | CT | Y | N | Y | Y | Y | N | Y | Simulation topic not given. |
| Ataee 2019 | Y | CT | Y | N | Y | Y | Y | N | Y | Groups matched by gender and age |
| Cao 2015 | Y | CT | Y | CT | CT | Y | Y | N | Y | Intervention was combined with practice evaluation. |
| Craig 2021 | Y | CT | Y | N/Y | Y | Y | Y | N | Y | Randomisation done by Programme Manager – blinded. Control group had simulation teaching in Week 4. |
| Dery 2019 | Y | Y | Y | N | Y | Y | Y | N | Y | Very low cost low tech simulation used. |
| Guerrero 2021 | Y | Y | Y | CT/N | CT | Y | Y | N | Y | Online written examination for group A had technical difficulties that might have affected final exam results |
| Hansen 2017 | Y | CT | N | CT | CT | Y | Y | N | Y | Crossover trial – time 1 results used only. Inter-rater reliability assessed with Kappa scores. |
| Harris 2011 | Y | CT | CT | CT | CT | CT | Y | N | Y | Unclear whether a true RCT as control group had already completed the course. |
| Hayden 2014 | Y | Y | Y | N | Y | Y | Y | N | Y | Large national multi-site trial with individual randomization. |
| Lee 2023 | Y | Y | Y | CT | Y | Y | Y | N | Y | Stated that “experimental and control groups were prevented from meeting each other by adjusting the timing of the intervention and data collection” but unclear how successful that was. |
| Luctkar-Flude 2012 | Y | CT | Y | CT | CT | Y | Y | N | Y | Comparison group was community volunteers rather than patients. |
| Olaussen 2022 | Y | Y | N | CT | Y | Y | Y | N | Y | Unclear why there was a large dropout rate in the control group. |
| Radhakrishnan 2007 | Y | CT | Y | CT | CT | Y | Y | N | Y | Very small RCT |
| Schlairet 2010 | Y | CT | N | CT | Y | Y | Y | N | Y | Crossover trial - time 1 results used only |
| Sears 2010 | Y | CT | CT | CT | CT | CT | Y | N | Y | Used Poisson distribution for outcome (number of medication errors) |
| Soccio 2017 | Y | CT | Y | N | CT | Y | Y | N | Y | Very little comparative information between groups |
| Son 2020 | Y | CT | Y | CT | Y | Y | Y | N | Y | Those with insufficient literacy skills were excluded from study, but it is unclear why people with poor literary skills are on a nursing course |
| Tawalbeh 2020 | Y | Y | Y | CT | Y | Y | Y | N | Y | Both groups also had lectures |
| Terzioğlu 2016 | Y | CT | Y | CT | CT | Y | Y | N | Y | Randomisation based on class attendance sequence |
| Witt 2018 | Y | Y | Y | CT | CT | Y | Y | N | Y | No demographic information comparing cases to controls |
| Yu 2017 | Y | CT | Y | Y | Y | Y | Y | N | Y | Both groups also had lectures |

Responses are Y=yes, CT=can’t tell, N=no

Q1: Did the trial address a clearly focused issue?

Q2: Was the assignment of patients to treatments randomized?

Q3: Were all of the patients who entered the trial properly accounted for at its conclusion?

Q4: Were patients, health workers and study personnel ‘blind’ to treatment?

Q5: Were the groups similar at the start of the trial?

Q6: Aside from the experimental intervention, were the groups treated equally?

Q9: Can the results be applied to the local population?

Q10: Were all clinically important outcomes considered?

Q11: Are the benefits worth the harms and costs?

NB. Q7 (How large was the treatment effect?) and Q8 (How precise was the estimate of the treatment effect?) are reported in the results tables

**Table S3. Cohort study results**

|  | **C1** | **C2** | **C3** | **C4** | **C5** | **C6A** | **C6B** | **C7A** | **C7B** | **C10** | **C11** | **C12** | **Comment** |
| --- | --- | --- | --- | --- | --- | --- | --- | --- | --- | --- | --- | --- | --- |
| Guerrero 2023 | Y | Y | CT | Y | Y | CT | CT | Y | Y | Y | Y | Y | This is in effect 4 cohorts compared to each other as each subsequent year had increasing amounts of simulation teaching. Unclear why a random selection rather than whole cohort of each year were selected |
| Hall 2015 | Y | Y | CT | Y | Y | CT | CT | Y | Y | Y | Y | Y | Can’t tell why some students had simulation whereas others did not. |
| Meyer 2011 | Y | N | Y | Y | CT | CT | CT | Y | Y | CT | Y | Y | Somewhat complicated presentation of results. |
| Roberts 2022 | Y | Y | CT | Y | Y | CT | CT | Y | Y | Y | Y | Y | Recruitment of pre-covid cohort unclear – was this the whole cohort or a selected number to match the covid cohort? |

Responses are Y=yes, CT=can’t tell, N=no

C1: Did the study address a clearly focused issue?

C2: Did the authors use an appropriate method to answer their question?

C3: Was the cohort recruited in an acceptable way?

C4: Was the exposure accurately measured to minimize bias?

C5: Was the outcome accurately measured to minimize bias?

C6A: Have the authors identified all important confounding factors?

C6B: Have they taken account of the confounding factors in the design and/or analysis?

C7A: Was the follow up of subjects complete enough?

C7B: Was the follow up of subjects long enough?

C10: Do you believe the results?

C11: Can the results be applied to the local population?

C12: Do the results of this study fit with other available evidence?

NB. C8 (How large was the treatment effect?) and C9 (How precise was the estimate of the treatment effect?) are reported in the results tables

**Table S4. Case control study results**

|  | **CC1** | **CC2** | **CC3** | **CC4** | **CC5** | **CC6A** | **CC6B** | **CC9** | **CC10** | **CC11** | **Comment** |
| --- | --- | --- | --- | --- | --- | --- | --- | --- | --- | --- | --- |
| Banjo-Ogunnowo 2022 | Y | Y | Y | Y | Y | CT | CT | Y | Y | Y | Both groups also had classroom learning |
| Bai 2023 | Y | Y | Y | Y | Y | CT | CT | Y | Y | Y | Explicitly non-blinded so potential for assessment bias |
| Centrella-Nigro 2016 | Y | Y | Y | Y | Y | CT | CT | Y | Y | Y | Historical controls |
| Curl 2016 | Y | N | Y | Y | Y | CT | CT | Y | Y | Y | Volunteers for the experimental group could have introduced systemic bias |
| Guerrero 2022 | Y | N | CT | CT | Y | CT | CT | Y | Y | Y | Unclear how cases and controls selected |
| Hwang 2020 | Y | N | CT | CT | Y | CT | CT | Y | Y | Y | Unclear how cases and controls selected. Self-efficacy higher in control group. |
| Lee 2014 | Y | N | CT | CT | Y | Ct | CT | Y | Y | Y | Unclear how cases and controls selected |
| Mancini 2019 | Y | Y | Y | Y | Y | CT | CT | Y | Y | Y | Historical control group. Randomisation within intervention cohort but whole cohort results presented |
| O’Lynn 2014 | Y | N | Y | Y | Y | CT | CT | Y | Y | Y | Very small control group as half refused to be videoed so could not be assessed |
| Raman 2019 | Y | N | Y | Y | Y | CT | CT | Y | Y | Y | Intervention group had higher grade point average at the start. |
| Reaves 2020 | Y | Y | Y | Y | Y | CT | CT | Y | Y | Y | Only the accelerated group comparison used as traditional cohort was not an appropriate control |
| Reid 2020 | Y | N | Y | Y | Y | CT | CT | Y | Y | Y | Blinding of investigator to group allocation during outcome assessment |
| Seo 2021 | Y | N | Y | Y | Y | CT | CT | Y | Y | Y | Control group had higher GPA at start |
| Thomas 2022 | Y | N | CT | Y | Y | CT | CT | Y | Y | Y | Simulation course was an elective but unclear why students opted for this elective |
| White 2021 | Y | Y | Y | Y | Y | CT | CT | Y | Y | Y | Historical control group, no demographic information comparing cases to controls |
| Woda 2019 | Y | Y | Y | Y | Y | CT | CT | Y | Y | Y | Historical control group, no demographic information comparing cases to controls |
| Yu 2021 | Y | N | Y | Y | Y | CT | CT | Y | Y | Y | Both groups also had lectures |

Responses are Y=yes, CT=can’t tell, N=no

CC1: Did the study address a clearly focused issue?

CC2: Did the authors use an appropriate method to answer their question?

CC3: Were the cases recruited in an acceptable way?

CC4: Were the controls selected in an acceptable way?

CC5: Was the exposure accurately measured to minimise bias?

CC6A: What confounding factors have the authors accounted for?

CC6B: Have the authors taken account of the potential confounding factors in the design and/or in their analysis?

CC9: Do you believe the results?

CC10: Can the results be applied to the local population?

CC11: Do the results of this study fit with other available evidence?

NB. CC7 (How large was the treatment effect?) and CC8 (How precise was the estimate of the treatment effect?) are reported in the results tables
